# DGCR8 Acts as an Adaptor for the Exosome Complex to Degrade Double-Stranded Structured RNAs

**DOI:** 10.1016/j.molcel.2015.11.011

**Published:** 2015-12-17

**Authors:** Sara Macias, Ross A. Cordiner, Philippe Gautier, Mireya Plass, Javier F. Cáceres

**Affiliations:** 1 Medical Research Council Human Genetics Unit, Institute of Genetics and Molecular Medicine, University of Edinburgh, Western General Hospital, Edinburgh EH4 2XU, UK; 2 Department of Biology, Center for Computational and Applied Transcriptomics, University of Copenhagen, Ole Maaloes Vej 5, 2200 Copenhagen, Denmark

## Abstract

The Microprocessor complex (DGCR8/Drosha) is required for microRNA (miRNA) biogenesis but also binds and regulates the stability of several types of cellular RNAs. Of particular interest, DGCR8 controls the stability of mature small nucleolar RNA (snoRNA) transcripts independently of Drosha, suggesting the existence of alternative DGCR8 complex(es) with other nucleases to process a variety of cellular RNAs. Here, we found that DGCR8 copurifies with subunits of the nuclear exosome, preferentially associating with its hRRP6-containing nucleolar form. Importantly, we demonstrate that DGCR8 is essential for the recruitment of the exosome to snoRNAs and to human telomerase RNA. In addition, we show that the DGCR8/exosome complex controls the stability of the human telomerase RNA component (hTR/TERC). Altogether, these data suggest that DGCR8 acts as an adaptor to recruit the exosome complex to structured RNAs and induce their degradation.

## Highlights

• DGCR8 forms an alternative complex with the hRRP6-containing form of the exosome
• DGCR8 acts as an adaptor to recruit the exosome to target structured RNAs
• The DGCR8/hRRP6 complex also controls the stability of human telomerase RNA

## Graphical Abstract

**Figure:**
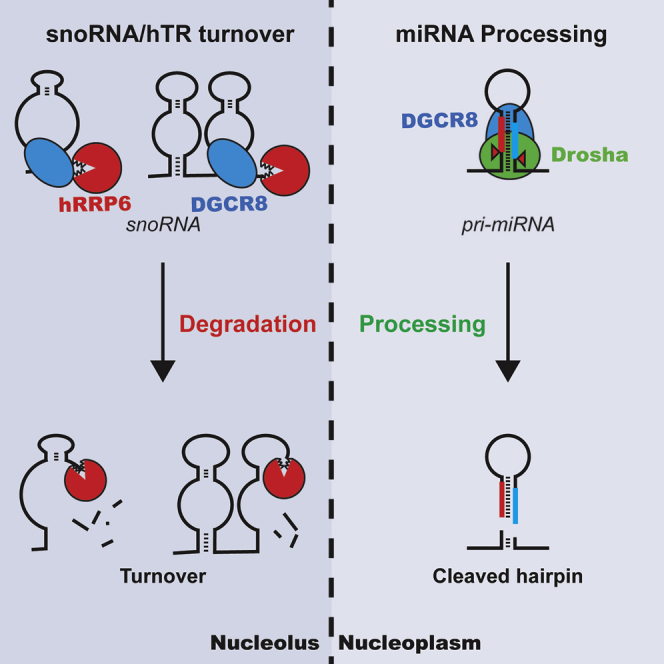

## Teaser

DGCR8 is part of the Microprocessor, a complex required for miRNA biogenesis. Macias et al. report that DGCR8 forms an alternative complex with the exosome, acting as an adaptor to recruit the exosome to mature snoRNAs and human telomerase RNA for degradation.

## Introduction

MicroRNAs are small noncoding RNAs that negatively regulate gene expression, influencing many biological processes (Ebert and Sharp, 2012). The nuclear step of miRNA biogenesis is catalyzed by the Microprocessor complex, comprising the RNase III enzyme Drosha and the double-stranded RNA-binding protein DGCR8 (DiGeorge critical region 8) and results in the production of precursor miRNAs (pre-miRNAs) (Denli et al., 2004, Gregory et al., 2004, Han et al., 2004). DGCR8 recognizes the RNA substrate in the nucleus through two double-stranded RNA binding motifs and acts as an anchor to direct the endonucleolytic cleavage by Drosha 11 base pairs-away from the base of the pri-miRNA hairpin (Nguyen et al., 2015). This processing event generates stem loop precursors (pre-miRNAs), which are exported to the cytoplasm by Exportin 5 and are further processed by Dicer into mature miRNAs (reviewed by Ha and Kim, 2014, Krol et al., 2010). The initial biochemical purification of Drosha revealed the existence of two different molecular weight complexes. A smaller complex, which is the minimally active catalytical complex, is composed of DGCR8 and Drosha and a larger complex containing several RNA-associated proteins including a subset of hnRNP proteins, DEAD box, and DEAH box family of RNA helicases and double-stranded RNA-binding proteins (Gregory et al., 2004, Siomi and Siomi, 2010).

Initially, noncanonical functions for the Microprocessor were suggested by the finding that a stem loop in the 5′UTR of the DGCR8 mRNA is bound and cleaved by the Microprocessor, in a negative feedback loop (Han et al., 2009, Kadener et al., 2009, Triboulet et al., 2009). A DGCR8 HITS-CLIP experiment revealed that, in addition to pri-miRNAs, the Microprocessor binds to a large number of structured RNAs that harbor a predicted secondary structure resembling that of a pri-miRNA. These include several hundred mRNAs, long intergenic noncoding RNAs (lincRNAs), small nucleolar RNAs (snoRNAs), and transposable elements, including LINE-1 elements (Heras et al., 2013, Macias et al., 2012). We previously found that the stability of snoRNAs is controlled in a DGCR8-dependent but Drosha-independent manner, suggesting the existence of an alternative DGCR8 complex, whereby its association with a yet-unidentified nuclease(s) could regulate the stability of this subset of RNAs (Macias et al., 2012).

The major RNA decay machinery in eukaryotes is the exosome complex that plays an important role in the processing and degradation of RNAs, both in the nucleus and in the cytoplasm and is functionally regulated by accessory factors (Mitchell et al., 1997; reviewed by Chlebowski et al., 2013, Houseley et al., 2006, Lorentzen et al., 2008). In yeast, this complex mediates the processing and controls steady-state levels of rRNA, snoRNAs, antisense RNAs, and cryptic unstable transcripts (Lykke-Andersen et al., 2011, Sloan et al., 2012). By contrast, the functions of this complex in higher eukaryotes are less well characterized. In humans, the nuclear form of the inactive core exosome is composed by nine different subunits, which form a ring structure and associate to two different catalytical subunits hDIS3 and hRRP6 (also called PM/SCl-100) (Houseley and Tollervey, 2008, Liu et al., 2006, Lykke-Andersen et al., 2011). Human DIS3 localizes in the nucleoplasm and acts as an endo/exonuclease, whereas the exonuclease hRRP6 concentrates mainly in the nucleolus (Lebreton et al., 2008, Tomecki et al., 2010). Additional associated factors have been proposed to confer substrate specificity to this machinery. The TRAMP complex, initially discovered in yeast, targets noncoding and aberrant RNAs by addition of a short noncanonical poly(A) tail, which in turn directs these transcripts for exosomal degradation (LaCava et al., 2005, Vanácová et al., 2005, Wyers et al., 2005). In humans, the trimeric Nuclear Exosome Targeting (NEXT) complex acts to recruit the exosome to promoter-upstream transcripts (PROMPTs) and to actively transcribed RNA-polymerase II transcripts (Lubas et al., 2011). It also promotes a functional link of the exosome with the cap bind complex (CBC) that is essential for transcription termination (Andersen et al., 2013). Here, we set out to identify the DGCR8 complex that is responsible for the Drosha-independent degradation of mature snoRNAs. We used mass spectrometry analysis of DGCR8 immunoprecipitations and found that DGCR8 interacts with components of the nuclear exosome, in particular the catalytically active hRRP6 subunit in the nucleolus. Importantly, we confirmed that DGCR8 is essential for the recruitment of the exosome to a particular subset of nucleolar transcripts, such as snoRNAs. Interestingly, we also show that the DGCR8/exosome complex, but not the canonical Microprocessor, controls the stability of another small transcript, the human telomerase RNA component (hTR/TERC). Altogether, these data suggest the existence of an alternative DGCR8 complex, whereby DGCR8 acts as an adaptor to recruit the exosome to double-stranded structured RNAs and promote their degradation.

## Results

### DGCR8 Interacts with the Human RNA Exosome Complex

**Figure 1:**
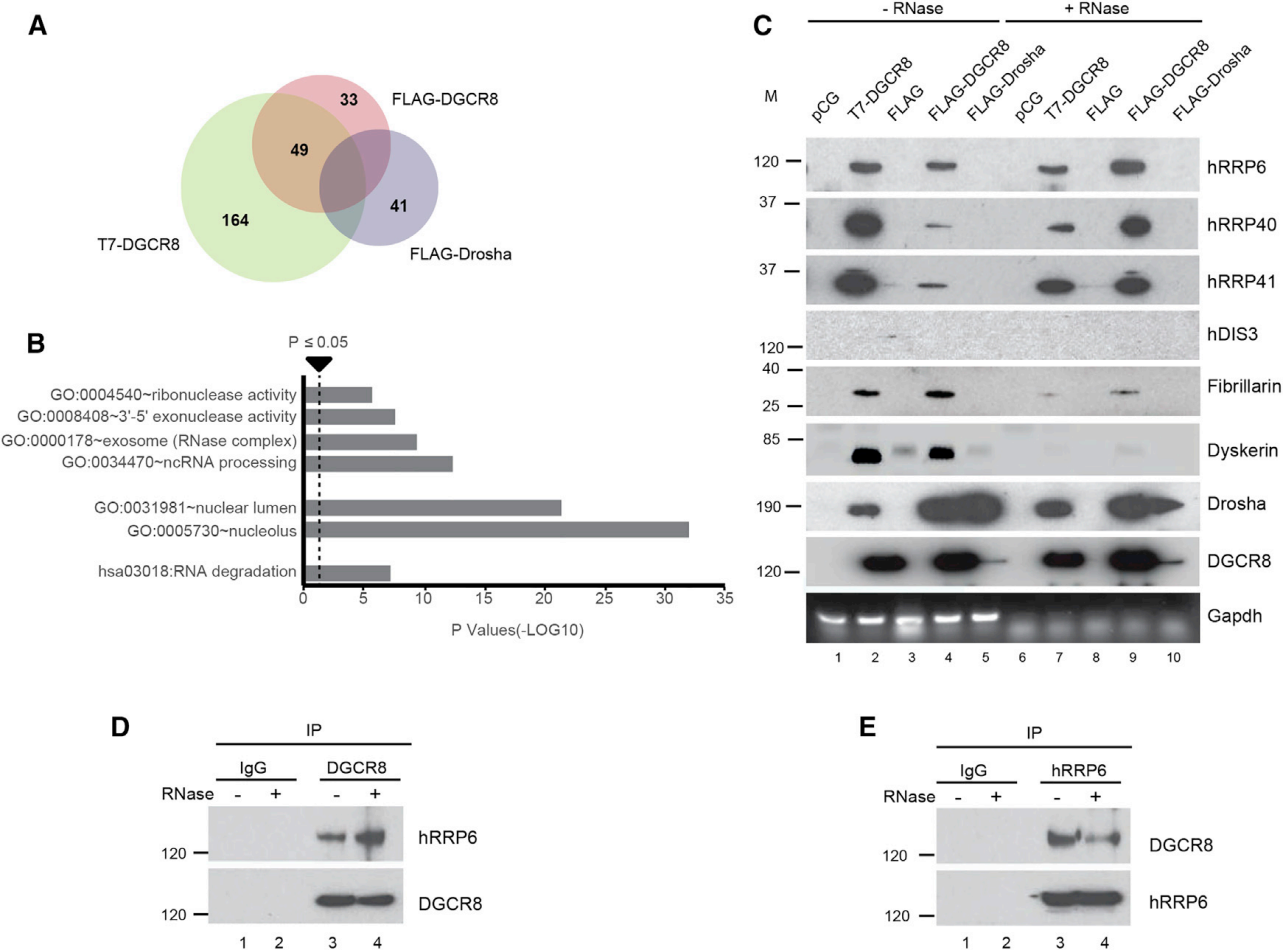
DGCR8 Interacts with the Exosome (A) Representation of the number of interacting partners identified by mass spectrometry (MS) analysis of immunoprecipitated T7-DGCR8, FLAG-DGCR8 and FLAG-Drosha using BioVenn (for a complete list of proteins identified by MS analyses, see Table S1; for DGCR8-exclusive interacting partners, see Figure S1B). (B) Gene ontology analyses of the 49 DGCR8-exclusive interacting partners (vertical dashed line represents significance, p ≤ 0.05). (C) Validation of proteins interacting with T7-DGCR8, FLAG-DGCR8, and FLAG-Drosha by immunoprecipitation followed by western blot analysis with specific antibodies, in the presence (lanes 6–10) or absence of RNase A (lanes 1–5). The RT-PCR amplification of Gapdh serves as a control for RNase treatment (bottom panel). (D and E) Reciprocal analysis of coimmunoprecipitated DGCR8 and hRRP6 endogenous proteins by western blot analysis with specific antibodies, in the presence (lanes 2 and 4) or absence of RNase A (lanes 1 and 3).

In order to identify DGCR8-interacting proteins, we performed immunoprecipitation (IP) coupled to mass spectrometry (MS) analysis of two different tagged versions of DGCR8 (FLAG and T7) in parallel with Drosha (FLAG) and their respective controls. Whole lanes from the immunoprecipitations were sent for MS analysis and a representative gel of the purification is shown on Figure S1A (available online). In order to define exclusive DGCR8-interacting partners, we selected those proteins that interacted with both tagged versions of DGCR8 and subtracted those that also interacted with Drosha (see Supplemental Experimental Procedures). This resulted in a total of 49 proteins that were exclusive to DGCR8 purification (Figures 1A and S1B; for a complete list, see Table S1). Gene ontology analyses revealed a significant enrichment for nucleolar-associated proteins and RNA processing factors, including proteins involved in RNA degradation and with exonuclease activity (Figure 1B). Strikingly, this analysis revealed that five out of the nine core subunits of the RNA exosome as well as the catalytical component hRRP6, a 3′-5′ exoribonuclease, interact with DGCR8 (see Figure S1B and Table S1). These results were validated with immunoprecipitations of the three overexpressed proteins (T7-DGCR8, FLAG-DGCR8, and FLAG-Drosha) in the presence or absence of RNases, followed by western blot analysis with specific antibodies. We confirmed the interaction of DGCR8 with hRRP6 and with subunits of the core exosome complex, hRRP40, and hRRP41 in an RNA-independent manner (Figure 1C). By contrast, the interactions with Fibrillarin, a component of the C/D snoRNP particle, and dyskerin, an H/ACA snoRNP factor ,were severely reduced in the presence of RNase, suggesting RNA-dependent interactions (Figure 1C, compare lanes 2 and 4 with lanes 7 and 9). Importantly, the other catalytical subunit that can associate with the nuclear exosome, hDIS3, could not be immunoprecipitated with DGCR8, confirming our MS analyses and suggesting that DGCR8 interacts with the nucleolar form of the exosome complex that is associated to hRRP6. Remarkably, none of these proteins were immunoprecipitated by Drosha, except its canonical Microprocessor partner, DGCR8, confirming that these are bona fide DGCR8 interacting partners. Immunoprecipitations of endogenous DGCR8 in the presence of RNase confirmed its interaction with endogenous hRRP6 (Figure 1D, lane 4). In agreement, we also pulled down DGCR8 when endogenous hRRP6 was immunoprecipitated in an RNA-independent manner (Figure 1E, lane 4). Altogether, these data suggest that DGCR8 associates with the RNA exosome complex, and mostly with the nucleolar form that contains the hRRP6 exonuclease. Importantly, Drosha did not interact with this complex, demonstrating that DGCR8 can form different complexes with other nucleases.

### DGCR8 Forms Two Different Molecular Weight Complexes

**Figure 2:**
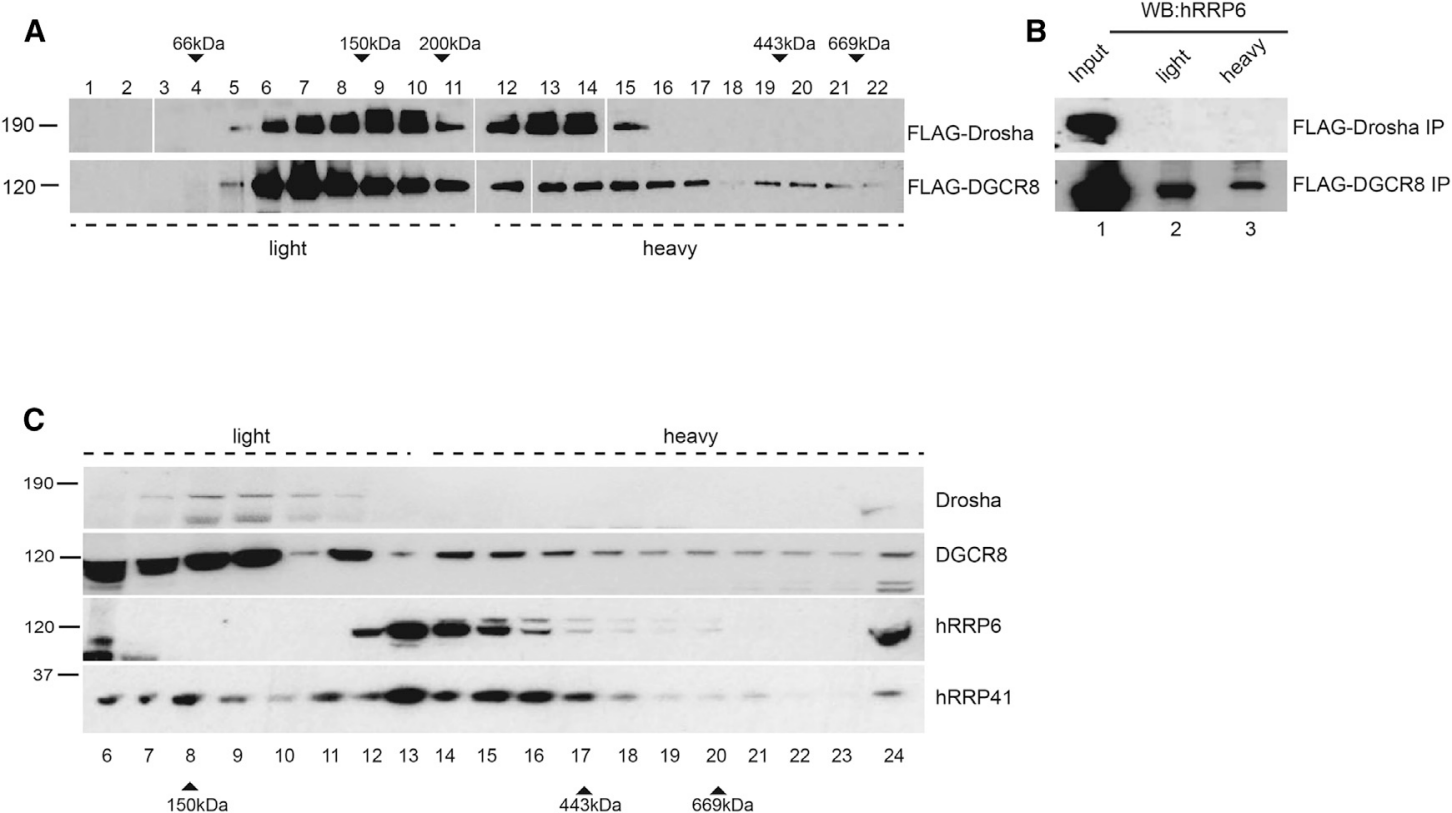
DGCR8 and the Exosome Coexist in a Complex (A) Sedimentation patterns of immunopurified FLAG-Drosha and FLAG-DGCR8 native complexes in 5%–30% glycerol gradient fractions, as revealed by western blot analysis with an anti-FLAG antibody. “Light” denotes lighter-molecular-weight fractions, whereas “heavy” indicates heavier molecular fractions. The migration of the molecular weight markers is indicated at the top (to see uncropped versions of these images, see Figure S2B). (B) Western blot of coimmunoprecipitated hRRP6 with FLAG-Drosha (top panel) and FLAG-DGCR8 (bottom panel) after glycerol gradient fractionation. Fractions from a 5%–30% glycerol gradient were pooled into light (lane 2), corresponding to fractions 1–11, and heavy (lane 3), corresponding to fractions 12–22, and run in a single lane for sensitivity purposes. (C) Sedimentation patterns of endogenous Drosha, DGCR8, hRRP6, and hRRP41 proteins in 5%–30% glycerol gradients from nuclear HEK293T cell extracts, as revealed by western blot analysis with specific antibodies. Lysates run in all gradients were produced in the presence of DNase and RNase.

The DGCR8 interactome analysis described above strongly suggests that DGCR8 may be part of at least two cellular complexes, one with Drosha to form the Microprocessor complex, and an additional complex with the exosome. In order to elucidate this possibility, we examined the sedimentation patterns of the native complexes formed by overexpressed DGCR8 and Drosha in 5%–30% glycerol gradients. For this purpose, immunoprecipitated FLAG-DGCR8 and FLAG-Drosha complexes were eluted under native conditions and loaded on the gradients. After centrifugation, the gradients were divided in twenty-two fractions and analyzed by western blot with specific antibodies. FLAG-Drosha immunoprecipitates were mainly present in fractions 6–15 (Figure 2A, top panel), whereas FLAG-DGCR8 sedimentation extended to heavier-molecular-weight fractions (fractions 16–22) (Figure 2A, bottom panel). We followed this observation by pooling light and heavy fractions and running them in two separate lanes. We confirmed that FLAG-Drosha coimmunoprecipitated DGCR8 in the light fractions (Figure S2A, lanes 8–10), but not hRRP6 (Figure 2B, upper panel). By contrast, FLAG-DGCR8 complexes were present in both light and heavy fractions together with hRRP6 (Figure 2B, lower panel), suggesting that hRRP6 can also form a high-molecular-weight complex with DGCR8, where Drosha is mostly absent. In addition, we also examined the sedimentation patterns of endogenous DGCR8, Drosha, and selected protein components of the exosome from HEK293T nuclear extracts in 5%–30% glycerol gradients. We observed that Drosha sedimented at the top of the gradient overlapping with DGCR8 (Figure 2C, fractions 7–11), whereas the exosome core component hRRP41 as well as hRRP6, the catalytic subunit of the exosome, cosedimented at fractions 12–17 (Figure 2C). Interestingly, DGCR8, but not Drosha, was also present in the heavier fractions, recapitulating the behavior of overexpressed DGCR8 (compare Figures 2C and 2A). In sum, these data are consistent with DGCR8 being part of at least two cellular complexes, a lighter molecular complex in association with Drosha, but also an association with hRRP6/exosome to form a heavier-molecular-weight complex.

### DGCR8 Interacts with hRRP6 in the Nucleolus

**Figure 3:**
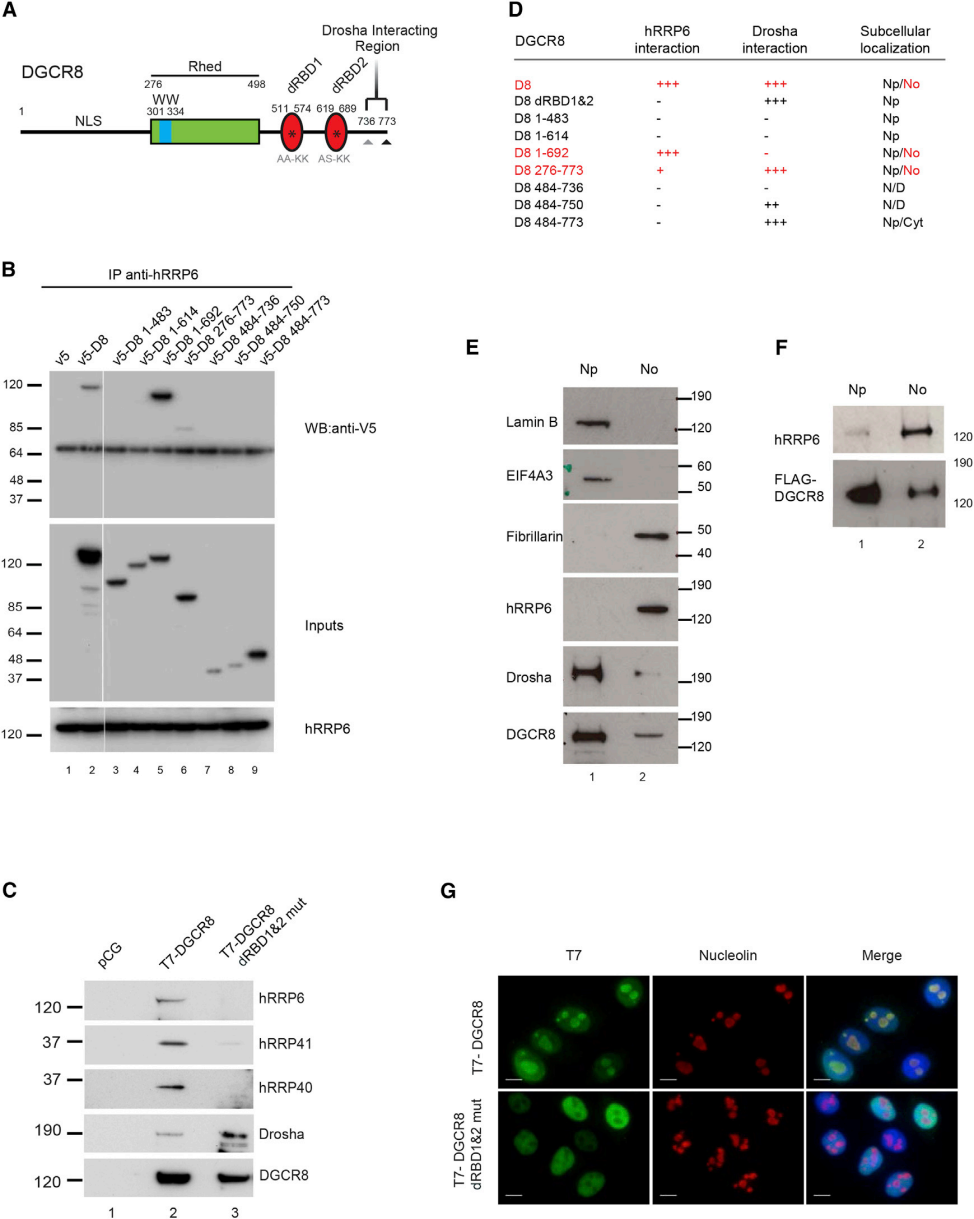
DGCR8 and hRRP6 Interact in the Nucleolus (A) Cartoon depicting the functional domains of DGCR8 (NLS, nuclear localization signal; Rhed, RNA-binding heme domain; WW, WW domain; dRBD1 and dRBD2, double-stranded RNA binding domain 1 and 2 and Drosha-interacting region). (B) Coimmunoprecipitation of V5-tagged wild-type DGCR8 (V5-D8, lane 2), empty plasmid as a negative control (V5, lane 1), and the indicated DGCR8 truncations (numbers represent amino acid positions, lanes 3–9). HEK293T cells transfected with these plasmids were subjected to immunoprecipitation of endogenous hRRP6 (bottom panel) followed by western blot with anti-V5 antibody (top panel). Inputs are shown in the middle panel. (C) HEK293T cells were transfected with plasmids overexpressing T7-tagged wild-type DGCR8 (T7-DGCR8) and a DGCR8 mutant with substitutions of critical residues that abrogate binding to dsRNA (AA-KK in dRBD1, and AS-KK in dRBD2 (as depicted in A) and subjected to anti-T7 immunoprecipitation followed by analyses of coimmunoprecipitated endogenous hRRP6, hRRP41, hRRP40, and Drosha proteins by western blot analysis. (D) Table summarizing interactions of mutant and truncated DGCR8 with hRRP6 (shown in B and C), Drosha (shown in C and in Figure S3), and their respective subcellular localizations, as previously characterized by Yeom et al., (2006). (E) Western blot analysis of the subcellular distribution of Drosha, DGCR8, and hRRP6 in nucleoplasmic (Np, lane 1) and nucleolar fractions (No, lane 2). Lamin B and eIF4AIII served as nucleoplasmic markers, whereas Fibrillarin is a nucleolar marker. (F) Western blot analysis of coimmunoprecipitated hRRP6 with FLAG-DGCR8 from nucleoplasmic (lane 1) and nucleolar fractions (lane 2). (G) Subcellular localization of transiently expressed T7-DGCR8 and T7-DGCR8 dRBD1&2 mut in HeLa cells. Nucleolin staining served as a nucleolar marker, whereas DAPI staining revealed nuclei.

In order to dissect the region in DGCR8 that is required for the interaction with hRRP6, we analyzed the interaction of seven V5-tagged deletion mutants of DGCR8 and also an additional mutation that abrogates the ability of DGCR8 to bind RNA (T7-DGCR8 dRBD1&2 mut) in transfected HEK293T cells (Figures 3A and 3D). Only full-length DGCR8 (v5-D8) and the D8 1–692 fragment, lacking the C-terminal region, could efficiently be coimmunoprecipitated with hRRP6 (Figure 3B, lanes 2 and 5). In addition, binding of hRRP6 to DGCR8 was barely detected with DGCR8 mutants that lacked the N-terminal region, where the NLS (nuclear localization signal) is located (D8 276–773, 484–736, 484–750, 484–773), as expected (Figure 3B, lanes 6–9). We also observed that the presence of the dsRNA binding motifs was required to efficiently coimmunoprecipitate endogenous hRRP6 (D8 1–483 and D8 1–614) (Figure 3B, lanes 3 and 4). This was confirmed by further evaluating a T7-DGCR8 construct that contained specific mutations in the dsRNA binding motifs (T7-DGCR8 dRBD1&2 mut), which are known to abolish RNA binding (AA-KK in dRBD1 and AS-KK in dRBD2, as described in Yeom et al., 2006). These point mutations abolished the interaction of DGCR8 not only with hRRP6, but also with other core components of the exosome, such as hRRP40 and hRRP41, but did not compromise interaction with Drosha (Figure 3C, lanes 2 and 3). These results show that whereas Drosha interacts with DGCR8 through the C-terminal region (Yeom et al., 2006; Figure S3), the interaction with the exosome requires an intact DGCR8 RNA-binding domain (Figure 3D). Next, we asked whether the two alternative DGCR8 complexes displayed a differential subcellular localization. We analyzed the endogenous intracellular distribution of hRRP6, DGCR8 and Drosha by western blot analysis following nucleoplasmic/nucleolar fractionation of HeLa cells. LaminB and eIF4AIII were used as nucleoplasmic markers, whereas Fibrillarin was used as a nucleolar maker (Figure 3E). We observed that hRRP6 was mainly present in the nucleolar fraction and Drosha was mainly nucleoplasmic, whereas DGCR8 was more abundant in the nucleoplasmic fraction but also present in the nucleolus (Figure 3E), suggesting that the interaction of DGCR8 and hRRP6 is restricted to the nucleolar compartment. This was confirmed by immunoprecipitation of transiently expressed FLAG-DGCR8 from the nucleoplasmic and nucleolar fractions, which revealed the preferential interaction of endogenous hRRP6 with DGCR8 in the nucleolar fraction (Figure 3F, lane 2). Immunofluorescence experiments revealed that wild-type T7-DGCR8 was present both in the nucleoplasm and nucleolus, as revealed by costaining with the nucleolar marker, nucleolin (Figure 3G, top panel), whereas a T7-DGCR8 dRBD1&2 mut, which does not interact with hRRP6, was mostly absent from the nucleolar compartment and preferentially localized to nucleoplasm (Figure 3G, bottom panel). Interestingly, the only DGCR8 mutant that was also able to efficiently coimmunoprecipitate endogenous hRRP6 (D8 1–692) also localizes to the nucleolus (Figure 3D). Furthermore, a bioinformatics prediction program (Scott et al., 2011) revealed a putative nucleolar localization signal overlapping with the second dsRBD motif in DGCR8, which is in agreement with the loss of nucleolar localization of a DGCR8 protein harboring a mutation in its dsRBD motifs (Figure 3G). Altogether this suggests that the mutually exclusive presence of two DGCR8 complexes is based on differential subcellular localization of the DGCR8 partners, Drosha and hRRP6, which are present within the nucleoplasm and nucleolus, respectively.

### DGCR8 Acts as an Adaptor for hRRP6 Recruitment to snoRNAs

**Figure 4:**
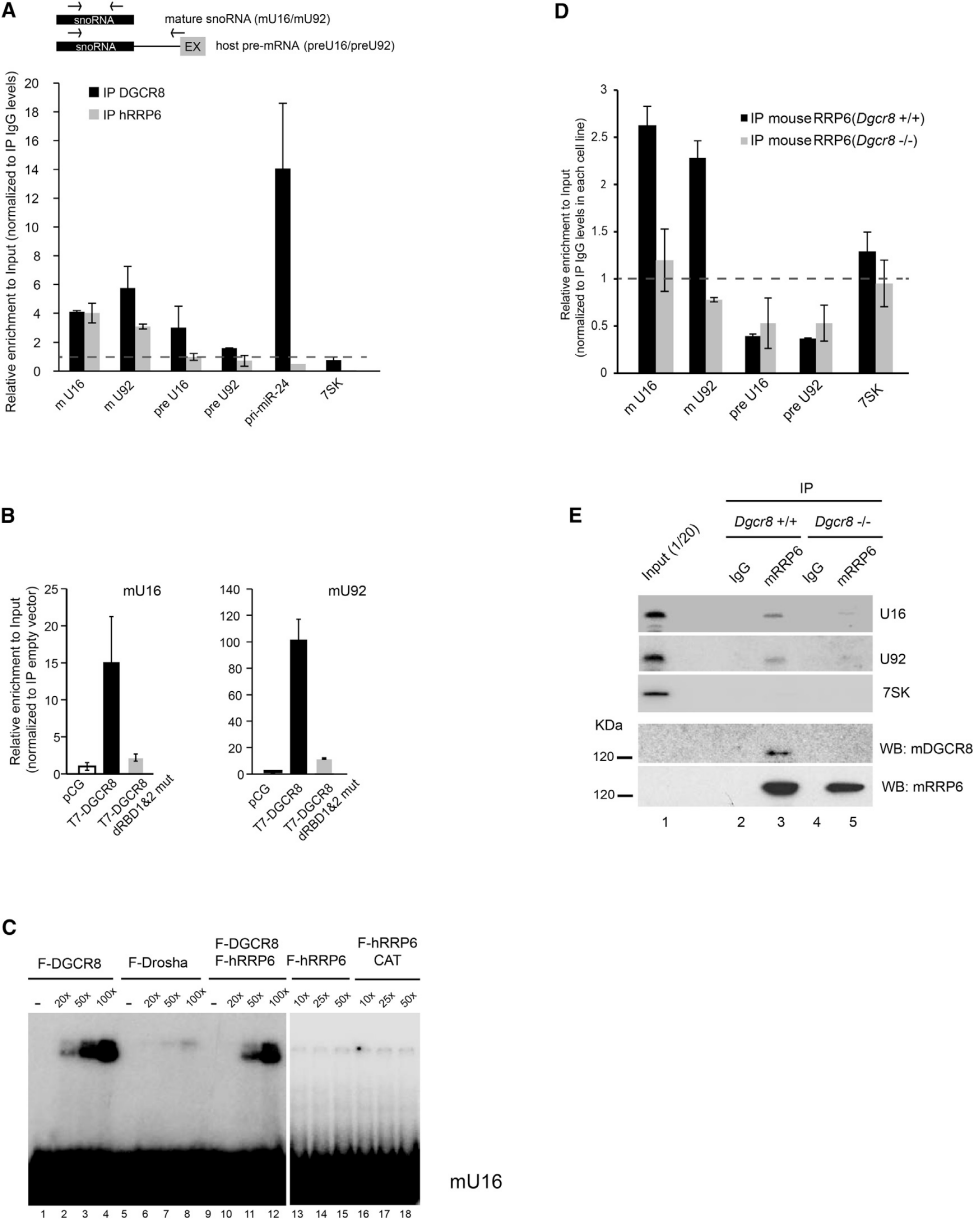
DGCR8 Promotes hRRP6 Binding to Mature snoRNAs (A) Schematic representation of primer pairs used to amplify mature snoRNAs (black box) or host pre-mRNAs (line represents the host intron) by qRT-PCR. Analysis of the associated mature snoRNAs (mU16 and mU92) and host pre-mRNAs (preU16 and preU92) to immunoprecipitated endogenous DGCR8 and hRRP6 in HEK293T cells. Pri-miR-24 serves as a positive control for a DGCR8 bound RNA and 7SK as a negative control. Values represented show at least the average of two different biological replicates ± SD. The enrichment of each RNA species is expressed relative to the levels of amplification in the control IgG immunoprecipitation (set to 1, represented as horizontal dashed line) and normalized to the levels in the Input material. (B) Analysis of the associated mature snoRNAs (mU16 and mU92, left and right panels, respectively) to overexpressed wild-type T7-DGCR8 and T7-DGCR8 dRBD1&2 mutant by qRT-PCR, using the same analysis as in (A). (C) EMSA analysis of mature U16 snoRNA in the presence of increasing amounts of purified FLAG-DGCR8, FLAG-Drosha, FLAG-hRRP6 and FLAG-hRRP6 CAT (D313N, catalytically dead mutant) (for purifications, see Figure S4B). The molar excess of protein versus radiolabeled RNA is indicated at the top of the panel (1× corresponds to 2.5 nM of protein and 2.5 nM of radiolabeled RNA). (D and E) Analysis of mouse RRP6 association to snoRNAs in mouse embryonic stem cells in the presence (*Dgcr8*^+/+^) or absence (*Dgcr8*^−/−^) of DGCR8 by qRT-PCR (D) and northern analyses (E). Quantitative RT-PCR data represent the average of at least two different biological replicates ± SD (D). The data were analyzed following the same procedure as in (A). Western blots for RRP6 immunoprecipitation levels and copurified DGCR8 are shown in (E) (bottom panels).

C/D box and H/ACA box snoRNAs associate to distinct sets of snoRNP proteins and guide two different modifications to the target RNAs, 2′-O-methylation and pseudouridylation, respectively (Tollervey and Kiss, 1997). They are mostly intronic and transcribed as part of the host gene, and following splicing of their host intron, their biogenesis involves trimming of the host introns from the 5′ and 3′ end. Subsequent release of the mature form of the snoRNA form is protected from further degradation by the core snoRNP components (Kiss, 2006). The identification of the exosome as a DGCR8 interacting partner in the nucleolus led us to ask whether DGCR8 could be acting as an adaptor for the recruitment of the exosome to snoRNAs. First, we tested binding of endogenous DGCR8 and hRRP6 to mature and precursor snoRNAs (host pre-mRNA) by immunoprecipitation followed by qRT-PCR analysis (IP-qRT-PCR) (Figure 4A). We observed that both DGCR8 and hRRP6 did indeed associate to a similar extent with two representative C/D and H/ACA mature snoRNA molecules (mU16 and mU92, respectively) (Figures 4A and S4A). DGCR8, but not hRRP6, associated to some extent to their host pre-mRNAs (preU16 and preU92) (Figure 4A), and as expected, we also detected binding of DGCR8, but not of hRRP6, to a canonical Microprocessor substrate (pri-miR-24) (Figure 4A). We confirmed that overexpressed DGCR8 associates to mature snoRNAs by IP-qRT-PCR, and that this binding is specific, since this association was abolished when using a mutant of DGCR8 that cannot longer bind dsRNA (T7-DGCR8 dRBD1&2 mut) (Figure 4B). In order to recapitulate these observations in vitro, we performed gel-shift assays with purified FLAG-tagged versions of DGCR8, Drosha, hRRP6 and a catalytically dead mutant of hRRP6 (D313N, (Januszyk et al., 2011)) expressed in HEK293T cells (see representative purification in Figure S4B). We observed that purified DGCR8 can directly bind to mature U16, although this binding was less efficient than to a canonical pri-miRNA (Figures 4C and S4C, lanes 1–4 compare molar ratios at the top of the panels); however, no binding was observed when using U1 snRNA as a negative control (Figure S4D). As expected, we only observed binding of Drosha to a canonical substrate, pri-miR-30c-1 (data not shown), but not to U16 (Figures 4C, lanes 5–9). The addition of FLAG-hRRP6, or a catalytically dead version of hRRP6, did not result in a shift (Figure 4C, lanes 13–18), and accordingly the combined addition of DGCR8 and hRRP6 did not obviously change the migration of the complex when compared to DGCR8 alone (Figure 4C, lanes 9–12). Taken together, these results suggest that DGCR8 could be the factor that enables hRRP6 binding to snoRNAs, acting as an adaptor protein to efficiently recruit the exosome complex to these species. In order to test this possibility, we immunoprecipitated endogenous RRP6 protein from wild-type mouse embryonic stem cells (*Dgcr8*^+/+^), or cells lacking DGCR8 (*Dgcr8*^−/−^) and analyzed RRP6 binding to mature snoRNAs by qRT-PCR and northern blot (Figures 4D and 4E, respectively). Importantly, we observed that mouse RRP6 binding to mature snoRNAs U16 and U92 was abrogated in the absence of DGCR8 (Figure 4D and Figure 4E, compare lanes 3 and 5). A similar result was obtained by immunoprecipitating overexpressed Flag-hRRP6 in *Dgcr8*^+/+^ and *Dgcr8*^−/−^ cells (Figures S4E and S4F). This shows that DGCR8 is essential to promote binding of hRRP6 to snoRNAs and suggests that DGCR8 acts as an adaptor to recruit the exosome complex to mature snoRNAs.

### DGCR8 and hRRP6 Control the Levels of Mature snoRNAs

**Figure 5:**
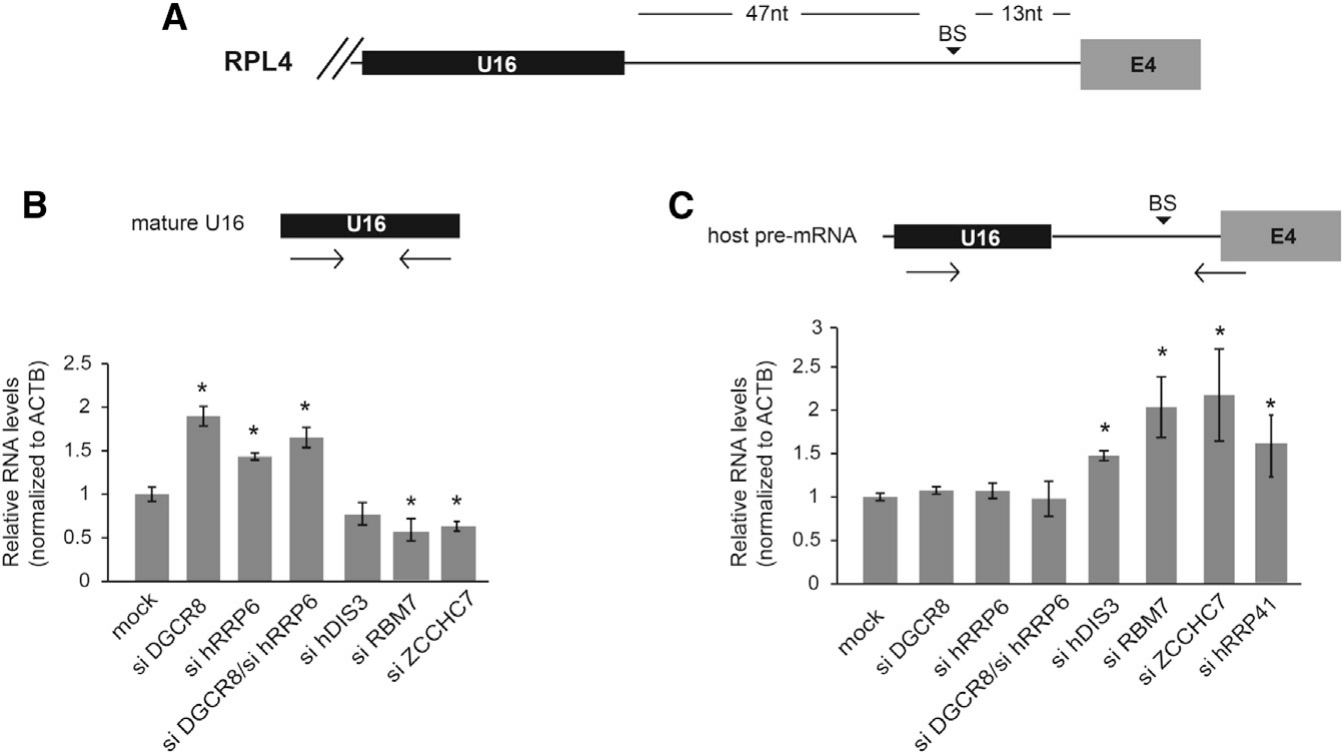
Depletion of DGCR8 and hRRP6 Specifically Stabilizes Mature snoRNAs (A) Schematic representation of U16 snoRNA location in intron 3 of the host *RPL4* pre-mRNA. BS, branch site; E4, exon 4. (B) HeLa cells were transiently depleted of DGCR8, hRRP6, hDIS3, RBM7, and ZCCHC7, and the levels of mature U16 were quantitated by qRT-PCR, using primers depicted on top of the panel. (C) Quantification of the host pre-mRNAs containing U16 snoRNA in HeLa cells depleted for all factors depicted in (B), but also including hRRP41. For levels of depletion in (B) and (C), see Figure S5D. All values represented in the two panels are the average of at least three biological replicas showing ± SEM. Asterisks denote significant p value (≤0.05) by Student’s t test.

The in vivo data presented above showed that DGCR8 is necessary to recruit hRRP6 to mature snoRNAs (Figure 4). Since both factors are in the same complex, their codepletion should not have additional effects on snoRNA levels. In order to investigate this, we quantified mature and precursor U16 snoRNAs by qRT-PCR upon transient depletion of these factors (see precursor U16 representation in Figure 5A). In agreement, depletion of human DGCR8 and hRRP6 in HeLa and SH-SY5Y cells resulted in a similar level of upregulation of mature U16 snoRNA, either when depleted alone or in combination, as shown (Figures 5B and S5B, respectively) or northern blot analyses (Figure S5A) (for depletion levels, see Figures S5D and S5E). Importantly, knockdown of other exosome-associated exonuclease factor hDIS3, a putative component of the human TRAMP complex, ZCCHC7, and a component of the NEXT complex, RBM7 (Lubas et al., 2011), which did not copurify with DGCR8, led to slight decrease of mature snoRNA levels, suggesting that these factors are not involved in the turnover of the mature form of this snoRNA, but rather processing (Figure 5B). In addition, we observed that the levels of the precursor host pre-mRNA, where U16 is contained, remained constant upon transient depletion of DGCR8 and hRRP6 (Figure 5C) that is in agreement with the fact that hRRP6 did not bind this transcript (Figure 4). By contrast, the transient depletion of hDIS3, RBM7, ZCCHC7 and a core component of the exosome, hRRP41, led to an accumulation of the U16 precursor (Figure 5C). This result, together with the observed decrease in the mature form of the snoRNAs (Figures 5B and S5A), suggests that both the exosome and these adaptor complexes are predominantly involved in the processing and maturation of precursor snoRNAs. Finally, we also observed upregulation of mature snoRNA levels, but not of the precursor forms, in mouse ESCs lacking DGCR8, confirming the effects observed in human cells (Macias et al., 2012) (Figure S5C). Altogether, these data strongly suggest that DGCR8 and hRRP6 form a cellular complex that controls mature snoRNA stability in vertebrates.

### SnoRNAs Are the Main Substrate of the DGCR8/hRRP6 Complex

**Figure 6:**
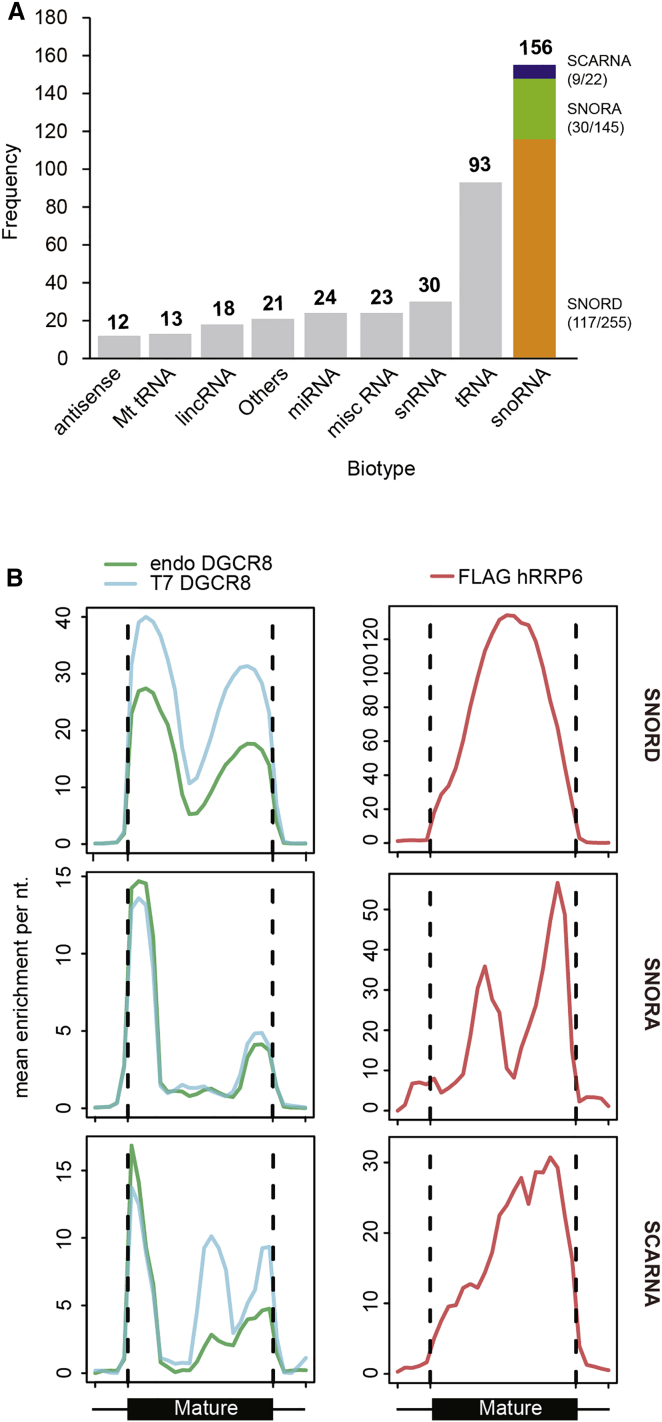
Genome-wide Identification of hRRP6 and DGCR8 Common RNA Substrates (A) Distribution of DGCR8 and hRRP6 overlapping significant clusters across ncRNAs loci. The colored column shows the distribution of snoRNA families bound by both DGCR8 and hRRP6. (B) Average read density of endogenous and overexpressed DGCR8 (left panels) and FLAG-hRRP6 CLIP experiments (right panels) over snoRNA genes and 5′/3′ flanking regions ± 50 nt.

In order to globally identify the substrates of the DGCR8/hRRP6 complex, we analyzed the overlap of the in vivo targets of the hRRP6 nuclease identified by iCLIP in HEK293Ts (manuscript in preparation) with the previously published HITS-CLIP targets of DGCR8 (Macias et al., 2012). Only significant clusters that overlapped from each CLIP experiment were considered as potential true common RNA substrates for the DGCR8/hRRP6 complex (see Supplemental Experimental Procedures). This resulted in the identification of 390 ncRNAs that were common to DGCR8 and hRRP6, with snoRNAs being the most overrepresented within this group (40% of the total common substrates, with 156 snoRNAs bound from the 422 of the expressed snoRNAs within HEK293T cells), followed by tRNAs (23% of total common substrates, represented by 93 different tRNAs) (Figure 6A). To assess the coverage of binding, we first identified which snoRNAs are expressed in HEK293T cells, using a previously published small RNA-seq data set (Kishore et al., 2013). From this set, we determined that both DGCR8 and hRRP6 bind to 117 out of 255 expressed C/D box snoRNAs (SNORD); 30 out of 145 expressed H/ACA box snoRNAs (SNORA), and 9 out of 22 expressed small Cajal RNAs (scaRNAs) (Figure 6A). Next, we calculated the average read density of CLIP tags from both hRRP6 and DGCR8 over snoRNA genes on a genome-wide context. We found that DGCR8 and hRRP6 CLIP reads fell mainly within the mature sequence of the snoRNAs and that this was common between all the snoRNA classes (Figure 6B).

So far, we have shown that the DGCR8/exosome complex is involved in the turnover of the mature form of U16 snoRNA (Figures 5B and S5A). Next, analysis of global snoRNA levels in RNA-seq data from cells depleted of DGCR8 or hRRP6 showed at least 19 commonly upregulated snoRNAs (Figure S6A), that were also confirmed by northern blot analyses (Figure S6B). Furthermore, we analyzed mature snoRNA levels in the absence of RRP6 using RNA-seq data from mouse ESCs that lack RRP6 (EXOSC10) gene expression (Pefanis et al., 2015). Interestingly, we observed that 64 of the mature snoRNAs expressed in this cell line, were at least 2-fold upregulated in the absence of RRP6 (Figure S6C). Altogether, these data reveal that both DGCR8 and hRRP6 can bind a wide range of mature snoRNAs, suggesting that the DGCR8/hRRP6 complex can regulate the abundance of mature snoRNAs on a global scale.

### DGCR8/hRRP6 Complex Controls hTR Stability and Telomere Length

**Figure 7:**
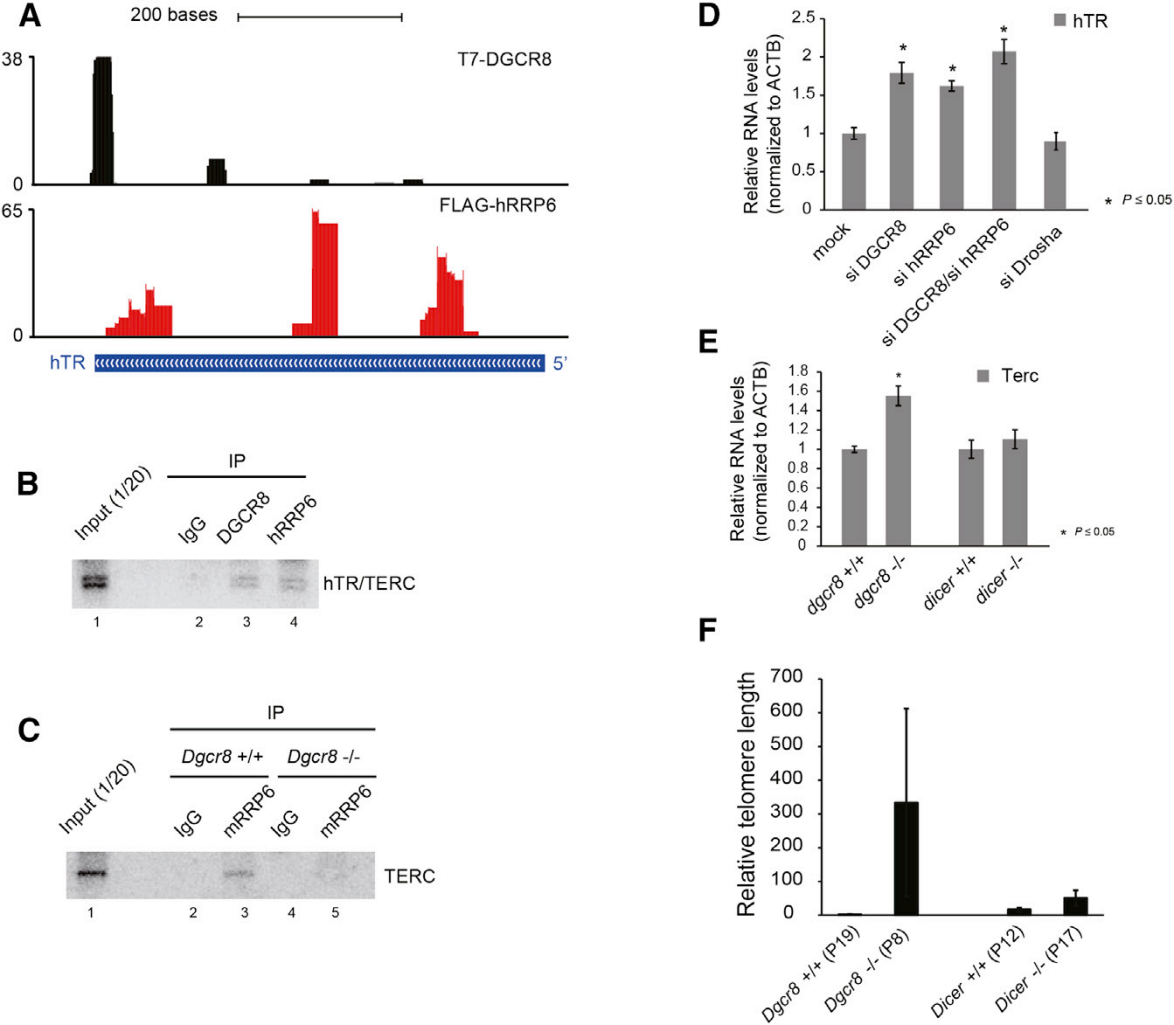
DGCR8/hRRP6 Complex Controls Human Telomerase RNA Levels (A) Distribution of DGCR8 and hRRP6 CLIP reads over hTR loci; numbers on the left represent number of reads obtained from each library mapping to hTR. (B and C) Northern analyses of associated hTR RNA with immunoprecipitated endogenous DGCR8 (lane 3) and hRRP6 (lane 4) in HEK293T cells (B) and with mouse RRP6 in the presence (*Dgcr8*^+/+^, lane 3) and absence (*Dgcr8*^−/−^, lane 5) of DGCR8 in mESC (C). (D) HeLa cells were transiently depleted of DGCR8, hRRP6 and Drosha and hTR levels were quantified by qRT-PCR (for depletion levels, see Figures S5D and S7B). (E) Levels of mouse TERC RNA were quantified by qRT-PCR in the absence of DGCR8 (*Dgcr8*^−/−^) and Dicer (*Dicer*^−/−^). All values represented in panels (D) and (E) are the average of at least three biological replicates ± SEM. Asterisks denote siginificant p value (≤0.05) by Student’s t test. (F) Relative telomere length quantification by qPCR of genomic DNA from cells lacking DGCR8 (*Dgcr8*^−/−^) and Dicer (*Dicer*^−/−^) and their respective wild-type controls (*Dgcr8*^+/+^ and *Dicer*^+/+^). Numbers in brackets represent the passage number. Absolute telomere quantification was normalized to a single-copy gene (c-*myc*), as described (Callicott and Womack, 2006). Values represent the average of three biological replicates ± SD.

CLIP data also revealed that both DGCR8 and hRRP6 bind to human telomerase RNA (hTR), with DGCR8 binding being mostly concentrated toward the 3′ end, where the H/ACA domain is located (Figure 7A). We validated the binding of endogenous DGCR8 and hRRP6 to hTR by Immunoprecipitation followed by northern blot analysis of telomerase RNA (Figure 7B). Next, we asked if DGCR8 was also acting as an adaptor to efficiently recruit the exosome to this particular RNA. For this purpose, we compared the amount of associated TERC RNA with RRP6 in the presence (*Dgcr8*^*+/+*^) or absence (*Dgcr8*^*−/−*^) of DGCR8. Similarly to other snoRNAs (Figure 4), the presence of DGCR8 was required to observe efficient coimmunoprecipitation of TERC RNA with RRP6 (Figure 7C, compare lanes 3 and 5). Importantly, depletion of DGCR8, hRRP6 or a combination of both resulted in hTR upregulation in HeLa cells as well as in SH-SY5Y cells (Figures 7D and S7A, respectively), whereas no changes in hTR levels were observed upon Drosha depletion (Figure 7D; for depletion levels, see Figure S7B). We also found that TERC levels remained constant in the absence of Dicer in ESCs (Figure 7E), in agreement with previous reports (Benetti et al., 2008); however, the absence of DGCR8 resulted in increased TERC levels (Figure 7E). These results suggest that the hTR transcript is a substrate of the DGCR8/hRRP6 complex and that in the absence of these components, hTR abundance is increased. Previous reports have suggested that increased expression of the hTR RNA is sufficient to boost telomerase activity in cultured cells (Cristofari and Lingner, 2006). Therefore, we hypothesized that the lack of DGCR8 should be enough to increase telomerase function, and this should result in an abnormal elongation of the telomeres. To test this, we measured the relative telomere length from mouse cells deficient in DGCR8 and Dicer using a qPCR based assay (Callicott and Womack, 2006). Notably, we observed a large increase in the telomeric qPCR signal in the absence of DGCR8, when compared to the parental cell line, but also in the absence of Dicer, which confirmed previous reports (Figure 7F) (Benetti et al., 2008). Alternatively, we used a dot blot assay to quantify the number of telomeric repeats (TTAGGG) of the same genomic samples, obtaining similar results (Figure S7C). All these data suggests that the DGCR8 alternative complex may have a role in controlling the number of telomeric repeats by regulating hTR levels.

## Discussion

Recent reports have suggested extended noncanonical functions for DGCR8 by describing its binding to a large number of cellular RNAs, which may adopt multiple RNA secondary structures (Macias et al., 2012, Macias et al., 2013, Roth et al., 2013). Remarkably, DGCR8 was shown to bind to a similar extent to precursor miRNAs and mature snoRNA molecules (29% versus 28% of the total DGCR8 binding sites in noncoding RNAs, respectively) and also to control the stability of C/D and H/ACA box snoRNAs in a Drosha-independent manner (Macias et al., 2012). Most human snoRNAs are located within introns, and their biogenesis is linked with the splicing of the host pre-mRNA (Hirose and Steitz, 2001, Hirose et al., 2003); thereafter a complex intranuclear trafficking directs most snoRNAs to the nucleolus and/or Cajal bodies (Kiss et al., 2006, Samarsky et al., 1998). Here, we describe a cellular complex that comprises DGCR8 and components of the nucleolar exosome that acts to control mature snoRNA and human telomerase RNA levels. The overlapping binding of DGCR8 and hRRP6 to both C/D and H/ACA box snoRNAs as well as to human telomerase RNA (hTR), demonstrated by independent CLIP experiments for these proteins, suggests a general role for the DGCR8/exosome complex in the regulation of snoRNA levels in the cell. In support of this, preliminary results show that the DGCR8/hRRP6 complex can regulate the abundance of mature snoRNAs on a global scale, as shown by the presence of 19 commonly upregulated snoRNAs (Figures 5B, S6A, and S6B). In the future, RNA-seq experiments with specially designed snoRNA libraries in cells depleted of DGCR8 or hRRP6 will help to determine the entire repertoire of cellular RNAs regulated by this complex.

The exosome core exclusively associates with hRRP6 in the nucleolus, whereas in the nucleoplasm is also associated to hDIS3 (Blüthner and Bautz, 1992, Tomecki et al., 2010). DGCR8 is present in the nucleoplasm but is also detectable in the nucleolar compartment, where it interacts with many nucleolar factors (Figure 3 and also see Shiohama et al., 2007). By contrast, Drosha is predominantly nucleoplasmic, whereas hRRP6 is highly enriched in the nucleolus (Allmang et al., 1999a, Blüthner and Bautz, 1992, Ge et al., 1992, Tomecki et al., 2010). Here, we show that DGCR8 can only interact with the exonuclease hRRP6 and the core exosome, when it is located within the nucleolus. We envision a scenario where the canonical DGCR8-containing Microprocessor complex processes pri-miRNAs in the nucleoplasm, whereas the alternative DGCR8-exosome complex targets and induces the degradation of mature snoRNAs following their transport to the nucleolus.

Recent characterization of Rrp6 targets in yeast showed enrichment for small, structured RNAs, such as tRNAs, snRNAs and snoRNAs. Of particular interest was the binding of yeast Rrp6 to the mature snoRNA snR40, which is suggestive of a role for this component in the surveillance and degradation of mature snoRNAs (Schneider et al., 2012). However, yeast Rrp6 has been also shown to be involved in the final trimming of precursor snoRNAs (Allmang et al., 1999b, van Hoof et al., 2000), suggesting a role for Rrp6 both in snoRNA biogenesis, as well as in decay. By contrast, the role of hRRP6 in snoRNA biogenesis and decay has not been characterized in humans, where it is only known to be important for the maturation of 5.8S and 18S rRNA, as well as for the decay of histone mRNAs and PROMPTs (Mullen and Marzluff, 2008, Schilders et al., 2007, Sloan et al., 2013). This is most likely to be an hRRP6-dependent but DGCR8-independent function, since mouse *Dgcr8*^−/−^ ESCs do not display any significant defect in rRNA biogenesis (Wang et al., 2007) or histone mRNA levels (data not shown). Our results suggest that, at least for the snoRNAs studied here, hRRP6 is not involved in snoRNA biogenesis, as it was described in yeast, rather it acts in concert with DGCR8 to specifically control mature snoRNA levels but not their precursors. This difference could also be explained by the absence of a DGCR8 homolog in yeast and the larger repertoire of ancillary proteins available to deal with the complexity of RNAs in higher order eukaryotes. Only recently, it was shown that human DIS3 is the main snoRNA-processing enzyme, whereas it was also suggested that RRP6 rather controls the levels of mature snoRNAs, as shown by northern blot analysis of a few selected snoRNAs (Szczepin et al., 2015).

Human telomerase is a ribonucleoprotein particle, containing the telomerase enzyme (TERT), which acts as a reverse transcriptase while the RNA component hTR/TERC serves as a template for the enzyme (Egan and Collins, 2012, Miracco et al., 2014). Analysis of the CLIP experiments for both DGCR8 (Macias et al., 2012) and hRRP6 (Figure 6) identified human telomerase RNA (hTR) as a putative substrate for this complex. The vertebrate telomerase RNA, TERC, contains a 3′ H/ACA snoRNA-like domain that binds H/ACA snoRNP proteins, which are essential to maintain correct levels of this RNA. Indeed, mutations impairing the function of these proteins lead to reduced hTR levels, which in turn results in poor telomere maintenance (Mitchell et al., 1999a, Vulliamy et al., 2008, Walne et al., 2007). Interestingly, this domain is only present in vertebrate organisms (Chen et al., 2000) and is important for localization (Jády et al., 2004) and maturation of the hTR (Mitchell et al., 1999b, Theimer et al., 2007). Human telomerase RNA is mainly localized in Cajal bodies, although a minor proportion can also be found in the nucleoli (Mitchell et al., 1999b). We observed that in the absence of DGCR8, mouse ESCs displayed an increase in TERC levels that were concomitant with an upregulation in the relative length of telomeres.

In summary, we have described here an alternative DGCR8 complex in association with the nucleolar form of the exosome. These data are compatible with a role for DGCR8 as an adaptor that acts to recruit and target the exosome for the degradation of mature snoRNAs and human telomerase RNA. This function may be especially relevant in vivo, where the exosome needs to be directed to different subclasses of RNA substrates by specific adaptor complexes in different subcellular compartments. Further research will be aimed to identify and characterize all the cellular targets of the DGCR8 alternative complex.

## Experimental Procedures

### Cell Lines, Transfections, and Antibodies

HEK293T, HeLa, and SH-SY5Y cells were grown under standard conditions in Dulbecco’s modified Eagle’s medium (DMEM). Mouse embryonic stem cells (mES) were grown on gelatin-coated plates (Sigma) without feeders in DMEM-high glucose supplemented with 15% (v/v) fetal bovine serum (GIBCO-Invitrogen), LIF, glutamine, and essential aminoacids. *Dgcr8*^−/−^ mES cells were purchased from Novus Biologicals (NBA1-19349) and the parental strain (v6.5) from Thermo Scientific (MES1402). *Dicer*^−/−^ and f/f Dicer were kindly provided by Robert Blelloch (UCSF) (Babiarz et al., 2008). Knock-down of endogenous proteins was performed in HeLa and SH-SY5Y cells after two rounds of siRNA transfection using Dharmafect 4 solution (Dharmacon). Briefly, cells were seeded in 6-well plates to 40% confluence and after 24 hr were transfected using 25 nM of each siRNA pool and 10 μl of the transfection reagent. The transfection medium was replaced after 24 hr and cells were grown for another 24 hr. Cells were then retransfected following the same protocol and collected 24 hr after the second transfection for analyses. siRNA pools were purchased from Dharmacon, Drosha (L-016996-00), DGCR8 (L-015713-00), hRRP6 (L-010904-00), hDIS3 (L-015405-01), hRRP41 (L-013760-00), ZCCHC7 (L-014804-01), RBM7 (L-017936-02), and nontargeting siRNAs (control) (D-001810-02). Overexpression analyses were performed in HEK293T cells by transfecting plasmids using Lipofectamine 2000 and following standard manufacturer’s protocol. Antibodies for immunoprecipitations and western blot analyses were the following, anti-DGCR8 antibody from Abcam (ab90579) and from Santa Cruz Biotech (sc-48473), anti-Drosha antibody from Novus Biologicals (NBP1-03349), anti-fibrillarin (ab4566), anti-hRRP6 (ab50558), anti-hRRP41 (ab137250) from Abcam. The anti-hRRP40 antibody (sc-98776) and anti-dyskerin (sc-48794) were purchased from Santa Cruz Biotech, and the anti-hDIS3 (14689-1-AP) antibody from Protein Tech. The anti-FLAG antibody was purchased from Sigma-Aldrich (F3165, mouse and F7425, rabbit), and the T7-antibody from Merck-Millipore (65922).

### Glycerol Gradients

Glycerol gradients (5%–30%) were poured using the Biocomp gradient station model 153 (BioComp Instruments, Inc., New Brunswick, Canada) and contained 50mM Tris (pH 0.5), 150 mM NaCl, and 1 mM EDTA. Nuclear HEK293T extracts or eluted FLAG immunoprecipitates were loaded in 11ml 5%–30% glycerol gradients and centrifuged for 16 hr at 41,000 rpms in a Sorvall SW41Ti rotor. Following centrifugation, fractions (500 μl) were collected manually from the top. When just showing heavy and light fractions from the gradient, the first 11 fractions were pooled and precipitated with TCA and the same applies for the next 11 fractions. All fractions were precipitated using standard TCA precipitation, the pellet was resuspended in loading buffer and analyzed in Tris-Glycine 4%–12% gels.

## Author Contributions

S.M., R.A.C., and J.F.C. conceived, designed, and interpreted the experiments. S.M. and R.A.C. performed all the experiments and data analysis. M.P. performed all the bioinformatics analyses, except for Figure S6A (which was performed by P.G.). J.F.C. supervised the whole project. The manuscript was cowritten by all authors.

## Accession Numbers

The accession number for the sequencing data from FLAG-hRRP6 CLIP and for HeLa depletions is GSE73376.

## Supplemental Information

Document S1. Figures S1–S7, Table S2, and Supplemental Experimental Procedures

Table S1. Identification of Factors Interacting with DGCR8 and Drosha by Mass Spectrometry Analyses, Related to Figure 1

Document S2. Article plus Supplemental Information

## References

[bib1] Allmang C., Kufel J., Chanfreau G., Mitchell P., Petfalski E., Tollervey D. (1999). Functions of the exosome in rRNA, snoRNA and snRNA synthesis. EMBO J..

[bib2] Allmang C., Petfalski E., Podtelejnikov A., Mann M., Tollervey D., Mitchell P. (1999). The yeast exosome and human PM-Scl are related complexes of 3′--> 5′ exonucleases. Genes Dev..

[bib3] Andersen P.R., Domanski M., Kristiansen M.S., Storvall H., Ntini E., Verheggen C., Schein A., Bunkenborg J., Poser I., Hallais M. (2013). The human cap-binding complex is functionally connected to the nuclear RNA exosome. Nat. Struct. Mol. Biol..

[bib4] Babiarz J.E., Ruby J.G., Wang Y., Bartel D.P., Blelloch R. (2008). Mouse ES cells express endogenous shRNAs, siRNAs, and other Microprocessor-independent, Dicer-dependent small RNAs. Genes Dev..

[bib5] Benetti R., Gonzalo S., Jaco I., Muñoz P., Gonzalez S., Schoeftner S., Murchison E., Andl T., Chen T., Klatt P. (2008). A mammalian microRNA cluster controls DNA methylation and telomere recombination via Rbl2-dependent regulation of DNA methyltransferases. Nat. Struct. Mol. Biol..

[bib6] Blüthner M., Bautz F.A. (1992). Cloning and characterization of the cDNA coding for a polymyositis-scleroderma overlap syndrome-related nucleolar 100-kD protein. J. Exp. Med..

[bib7] Callicott R.J., Womack J.E. (2006). Real-time PCR assay for measurement of mouse telomeres. Comp. Med..

[bib8] Chen J.L., Blasco M.A., Greider C.W. (2000). Secondary structure of vertebrate telomerase RNA. Cell.

[bib9] Chlebowski A., Lubas M., Jensen T.H., Dziembowski A. (2013). RNA decay machines: the exosome. Biochim. Biophys. Acta.

[bib10] Cristofari G., Lingner J. (2006). Telomere length homeostasis requires that telomerase levels are limiting. EMBO J..

[bib11] Denli A.M., Tops B.B.J., Plasterk R.H., Ketting R.F., Hannon G.J. (2004). Processing of primary microRNAs by the Microprocessor complex. Nature.

[bib12] Ebert M.S., Sharp P.A. (2012). Roles for microRNAs in conferring robustness to biological processes. Cell.

[bib13] Egan E.D., Collins K. (2012). Biogenesis of telomerase ribonucleoproteins. RNA.

[bib14] Ge Q., Frank M.B., O’Brien C., Targoff I.N. (1992). Cloning of a complementary DNA coding for the 100-kD antigenic protein of the PM-Scl autoantigen. J. Clin. Invest..

[bib15] Gregory R.I., Yan K.-P., Amuthan G., Chendrimada T., Doratotaj B., Cooch N., Shiekhattar R. (2004). The Microprocessor complex mediates the genesis of microRNAs. Nature.

[bib16] Ha M., Kim V.N. (2014). Regulation of microRNA biogenesis. Nat. Rev. Mol. Cell Biol..

[bib17] Han J., Lee Y., Yeom K.-H., Kim Y.-K., Jin H., Kim V.N. (2004). The Drosha-DGCR8 complex in primary microRNA processing. Genes Dev..

[bib18] Han J., Pedersen J.S., Kwon S.C., Belair C.D., Kim Y.-K.K., Yeom K.-H.H., Yang W.-Y.Y., Haussler D., Blelloch R., Kim V.N. (2009). Posttranscriptional crossregulation between Drosha and DGCR8. Cell.

[bib19] Heras S.R., Macias S., Plass M., Fernandez N., Cano D., Eyras E., Garcia-Perez J.L., Cáceres J.F. (2013). The Microprocessor controls the activity of mammalian retrotransposons. Nat. Struct. Mol. Biol..

[bib20] Hirose T., Steitz J.A. (2001). Position within the host intron is critical for efficient processing of box C/D snoRNAs in mammalian cells. Proc. Natl. Acad. Sci. USA.

[bib21] Hirose T., Shu M.-D., Steitz J.A. (2003). Splicing-dependent and -independent modes of assembly for intron-encoded box C/D snoRNPs in mammalian cells. Mol. Cell.

[bib22] Houseley J., Tollervey D. (2008). The nuclear RNA surveillance machinery: the link between ncRNAs and genome structure in budding yeast?. Biochim. Biophys. Acta.

[bib23] Houseley J., LaCava J., Tollervey D. (2006). RNA-quality control by the exosome. Nat. Rev. Mol. Cell Biol..

[bib24] Jády B.E., Bertrand E., Kiss T. (2004). Human telomerase RNA and box H/ACA scaRNAs share a common Cajal body-specific localization signal. J. Cell Biol..

[bib25] Januszyk K., Liu Q., Lima C.D. (2011). Activities of human RRP6 and structure of the human RRP6 catalytic domain. RNA.

[bib26] Kadener S., Rodriguez J., Abruzzi K.C., Khodor Y.L., Sugino K., Marr M.T., Nelson S., Rosbash M. (2009). Genome-wide identification of targets of the drosha-pasha/DGCR8 complex. RNA.

[bib27] Kishore S., Gruber A.R., Jedlinski D.J., Syed A.P., Jorjani H., Zavolan M. (2013). Insights into snoRNA biogenesis and processing from PAR-CLIP of snoRNA core proteins and small RNA sequencing. Genome Biol..

[bib28] Kiss T. (2006). SnoRNP biogenesis meets Pre-mRNA splicing. Mol. Cell.

[bib29] Kiss T., Fayet E., Jády B.E., Richard P., Weber M. (2006). Biogenesis and intranuclear trafficking of human box C/D and H/ACA RNPs. Cold Spring Harb. Symp. Quant. Biol..

[bib30] Krol J., Loedige I., Filipowicz W. (2010). The widespread regulation of microRNA biogenesis, function and decay. Nat. Rev. Genet..

[bib31] LaCava J., Houseley J., Saveanu C., Petfalski E., Thompson E., Jacquier A., Tollervey D. (2005). RNA degradation by the exosome is promoted by a nuclear polyadenylation complex. Cell.

[bib32] Lebreton A., Tomecki R., Dziembowski A., Séraphin B. (2008). Endonucleolytic RNA cleavage by a eukaryotic exosome. Nature.

[bib33] Liu Q., Greimann J.C., Lima C.D. (2006). Reconstitution, activities, and structure of the eukaryotic RNA exosome. Cell.

[bib34] Lorentzen E., Basquin J., Conti E. (2008). Structural organization of the RNA-degrading exosome. Curr. Opin. Struct. Biol..

[bib35] Lubas M., Christensen M.S., Kristiansen M.S., Domanski M., Falkenby L.G., Lykke-Andersen S., Andersen J.S., Dziembowski A., Jensen T.H. (2011). Interaction profiling identifies the human nuclear exosome targeting complex. Mol. Cell.

[bib36] Lykke-Andersen S., Tomecki R., Jensen T.H., Dziembowski A. (2011). The eukaryotic RNA exosome: same scaffold but variable catalytic subunits. RNA Biol..

[bib37] Macias S., Plass M., Stajuda A., Michlewski G., Eyras E., Cáceres J.F. (2012). DGCR8 HITS-CLIP reveals novel functions for the Microprocessor. Nat. Struct. Mol. Biol..

[bib38] Macias S., Cordiner R.A., Cáceres J.F. (2013). Cellular functions of the microprocessor. Biochem. Soc. Trans..

[bib39] Miracco E.J., Jiang J., Cash D.D., Feigon J. (2014). Progress in structural studies of telomerase. Curr. Opin. Struct. Biol..

[bib40] Mitchell P., Petfalski E., Shevchenko A., Mann M., Tollervey D. (1997). The exosome: a conserved eukaryotic RNA processing complex containing multiple 3′-->5′ exoribonucleases. Cell.

[bib41] Mitchell J.R., Wood E., Collins K. (1999). A telomerase component is defective in the human disease dyskeratosis congenita. Nature.

[bib42] Mitchell J.R., Cheng J., Collins K. (1999). A box H/ACA small nucleolar RNA-like domain at the human telomerase RNA 3′ end. Mol. Cell. Biol..

[bib43] Mullen T.E., Marzluff W.F. (2008). Degradation of histone mRNA requires oligouridylation followed by decapping and simultaneous degradation of the mRNA both 5′ to 3′ and 3′ to 5′. Genes Dev..

[bib44] Nguyen T.A.A., Jo M.H.H., Choi Y.-G., Park J., Kwon S.C.C., Hohng S., Kim V.N.N., Woo J.-S. (2015). Functional anatomy of the human microprocessor. Cell.

[bib45] Pefanis E., Wang J., Rothschild G., Lim J., Kazadi D., Sun J., Federation A., Chao J., Elliott O., Liu Z.-P. (2015). RNA exosome-regulated long non-coding RNA transcription controls super-enhancer activity. Cell.

[bib46] Roth B.M., Ishimaru D., Hennig M. (2013). The core microprocessor component DiGeorge syndrome critical region 8 (DGCR8) is a nonspecific RNA-binding protein. J. Biol. Chem..

[bib47] Samarsky D.A., Fournier M.J., Singer R.H., Bertrand E. (1998). The snoRNA box C/D motif directs nucleolar targeting and also couples snoRNA synthesis and localization. EMBO J..

[bib48] Schilders G., van Dijk E., Pruijn G.J.M. (2007). C1D and hMtr4p associate with the human exosome subunit PM/Scl-100 and are involved in pre-rRNA processing. Nucleic Acids Res..

[bib49] Schneider C., Kudla G., Wlotzka W., Tuck A., Tollervey D. (2012). Transcriptome-wide analysis of exosome targets. Mol. Cell.

[bib50] Scott M.S., Troshin P.V., Barton G.J. (2011). NoD: a Nucleolar localization sequence detector for eukaryotic and viral proteins. BMC Bioinformatics.

[bib51] Shiohama A., Sasaki T., Noda S., Minoshima S., Shimizu N. (2007). Nucleolar localization of DGCR8 and identification of eleven DGCR8-associated proteins. Exp. Cell Res..

[bib52] Siomi H., Siomi M.C. (2010). Posttranscriptional regulation of microRNA biogenesis in animals. Mol. Cell.

[bib53] Sloan K.E., Schneider C., Watkins N.J. (2012). Comparison of the yeast and human nuclear exosome complexes. Biochem. Soc. Trans..

[bib54] Sloan K.E., Mattijssen S., Lebaron S., Tollervey D., Pruijn G.J.M., Watkins N.J. (2013). Both endonucleolytic and exonucleolytic cleavage mediate ITS1 removal during human ribosomal RNA processing. J. Cell Biol..

[bib55] Szczepin T., Kalisiak K., Tomecki R., Labno A., Borowski L.S., Kulinski T.M., Adamska D., Kosinska J., Dziembowski A. (2015). Dros. Inf. Serv.3 shapes the RNA polymerase II transcriptome in humans by degrading a variety of unwanted transcripts. Genome Biol..

[bib56] Theimer C.A., Jády B.E., Chim N., Richard P., Breece K.E., Kiss T., Feigon J. (2007). Structural and functional characterization of human telomerase RNA processing and cajal body localization signals. Mol. Cell.

[bib57] Tollervey D., Kiss T. (1997). Function and synthesis of small nucleolar RNAs. Curr. Opin. Cell Biol..

[bib58] Tomecki R., Kristiansen M.S., Lykke-Andersen S., Chlebowski A., Larsen K.M., Szczesny R.J., Drazkowska K., Pastula A., Andersen J.S., Stepien P.P. (2010). The human core exosome interacts with differentially localized processive RNases: hDros. Inf. Serv.3 and hDros. Inf. Serv.3L. EMBO J..

[bib59] Triboulet R., Chang H.-M., Lapierre R.J., Gregory R.I. (2009). Post-transcriptional control of DGCR8 expression by the Microprocessor. RNA.

[bib60] van Hoof A., Staples R.R., Baker R.E., Parker R. (2000). Function of the ski4p (Csl4p) and Ski7p proteins in 3′-to-5′ degradation of mRNA. Mol. Cell. Biol..

[bib61] Vanácová S., Wolf J., Martin G., Blank D., Dettwiler S., Friedlein A., Langen H., Keith G., Keller W. (2005). A new yeast poly(A) polymerase complex involved in RNA quality control. PLoS Biol..

[bib62] Vulliamy T., Beswick R., Kirwan M., Marrone A., Digweed M., Walne A., Dokal I. (2008). Mutations in the telomerase component NHP2 cause the premature ageing syndrome dyskeratosis congenita. Proc. Natl. Acad. Sci. USA.

[bib63] Walne A.J., Vulliamy T., Marrone A., Beswick R., Kirwan M., Masunari Y., Al-Qurashi F.-H., Aljurf M., Dokal I. (2007). Genetic heterogeneity in autosomal recessive dyskeratosis congenita with one subtype due to mutations in the telomerase-associated protein NOP10. Hum. Mol. Genet..

[bib64] Wang Y., Medvid R., Melton C., Jaenisch R., Blelloch R. (2007). DGCR8 is essential for microRNA biogenesis and silencing of embryonic stem cell self-renewal. Nat. Genet..

[bib65] Wyers F., Rougemaille M., Badis G., Rousselle J.-C., Dufour M.-E., Boulay J., Régnault B., Devaux F., Namane A., Séraphin B. (2005). Cryptic pol II transcripts are degraded by a nuclear quality control pathway involving a new poly(A) polymerase. Cell.

[bib66] Yeom K.-H.H., Lee Y., Han J., Suh M.R., Kim V.N. (2006). Characterization of DGCR8/Pasha, the essential cofactor for Drosha in primary miRNA processing. Nucleic Acids Res..

