# Supplementary material for: DGCR8 Acts as an Adaptor for the Exosome Complex to Degrade Double-Stranded Structured RNAs
Source: Mol Cell. 2015 Dec 17;60(6):873–85. doi: 10.1016/j.molcel.2015.11.011 (PMC4691244; doi:10.1016/j.molcel.2015.11.011)
Supplement: Document S1. Figures S1–S7, Table S2, and Supplemental Experimental Procedures [file mmc1.pdf]

Molecular Cell, Volume *60*

Supplemental Information

DGCR8 Acts as an Adaptor for the Exosome Complex to Degrade Double-Stranded Structured RNAs

Sara Macias, Ross A. Cordiner, Philippe Gautier, Mireya Plass, and Javier F. Cáceres

Figure S1

A

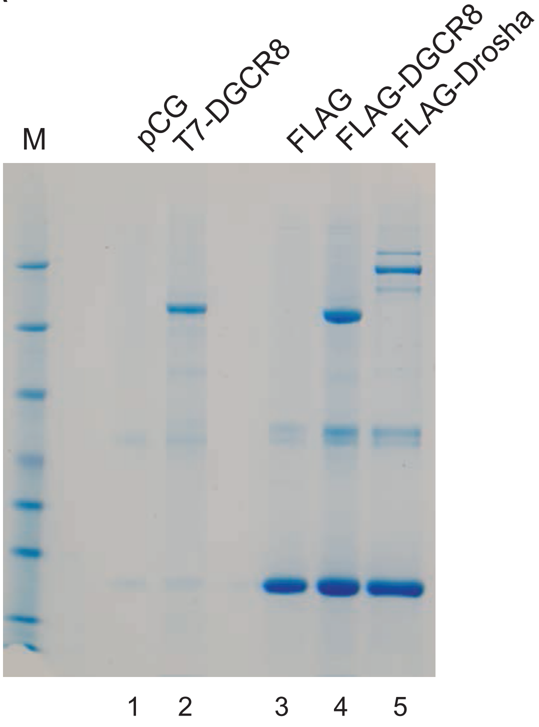

B

List of DGCR8-exclusive associated proteins

ALYREF
BRIX1
C7orf50
CDC5L
CMSS1
DDX21
DHX30
DIMT1
EBNA1BP2
EIF2S3
EIF6
EXOSC10 (hRRP6)
EXOSC2 (hRRP4)
EXOSC3 (hRRP40)
EXOSC4 (hRRP41)
EXOSC6 (hMTR3)
EXOSC9 (hRRP45)
GNL3
GRWD1
GTPBP4
H1FX
H3F3C
HIST1H1C
HIST1H2BJ
IGF2BP1
ILF3
LUC7L
LYAR
MINA
MMTAG2
MYBBP1A
NIFK
NOP2
NPM1
PURA
RBM28
RBM34
RPF2
RSBN1L
RSL1D1
SDAD1
SRP14
SRP72
SRP9
SSB
STAU1
TRMT1L
UPF3B
XRN2

Figure S1, related to Figure 1. Identification of DGR8 interacting partners by mass spectrometry. (A) Mass spectrometry analyses was carried out on immunoprecipitated T7-DGCR8, FLAG-DGCR8 and FLAG-Drosha (lanes 2, 4 and 5). As a negative control, extracts of cells transfected with control plasmids were immunoprecipitated using T7 (pCG, lane 1) and FLAG antibody coated beads (FLAG control plasmid, lane 3). (B) List of common proteins exclusively co-purified with T7-DGCR8 and FLAG-DGCR8. The list of DGCR8-exclusive interacting partners comprises 49 proteins and includes the six subunits of the eukaryotic exosome that are highlighted by a red box.

Figure S2

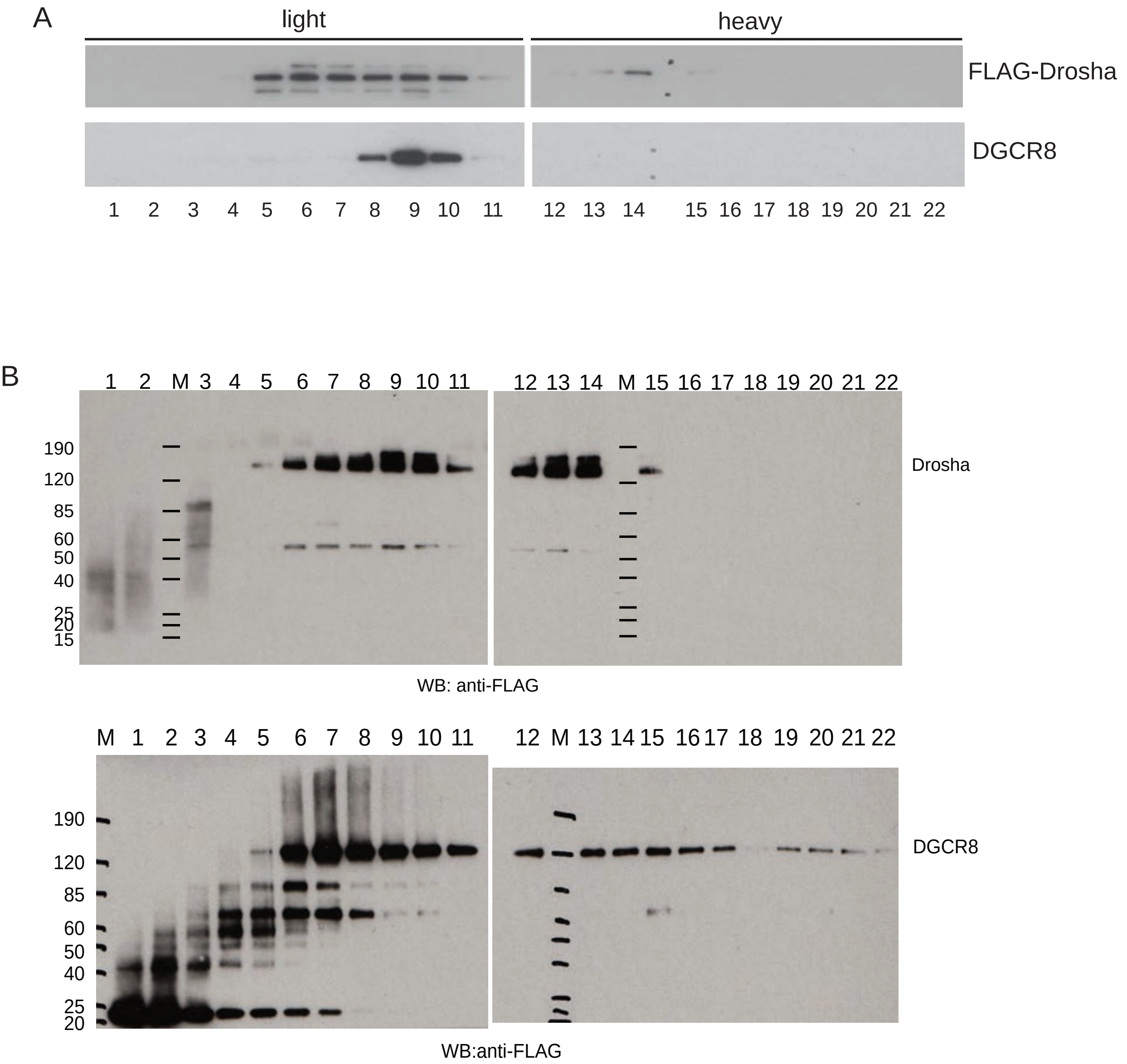

Figure S2, related to Figure 2. Differential sedimentation pattern of native complexes associated with DGCR8 and Drosha. (A) Western-blot analyses of immunoprecipitated FLAG-Drosha native complexes in 5-30% glycerol gradient fractions (upper panel) and the co-associated DGCR8 protein (lower panel). (B) Uncropped versions of western-blots shown in Figure 2A.

Figure S3

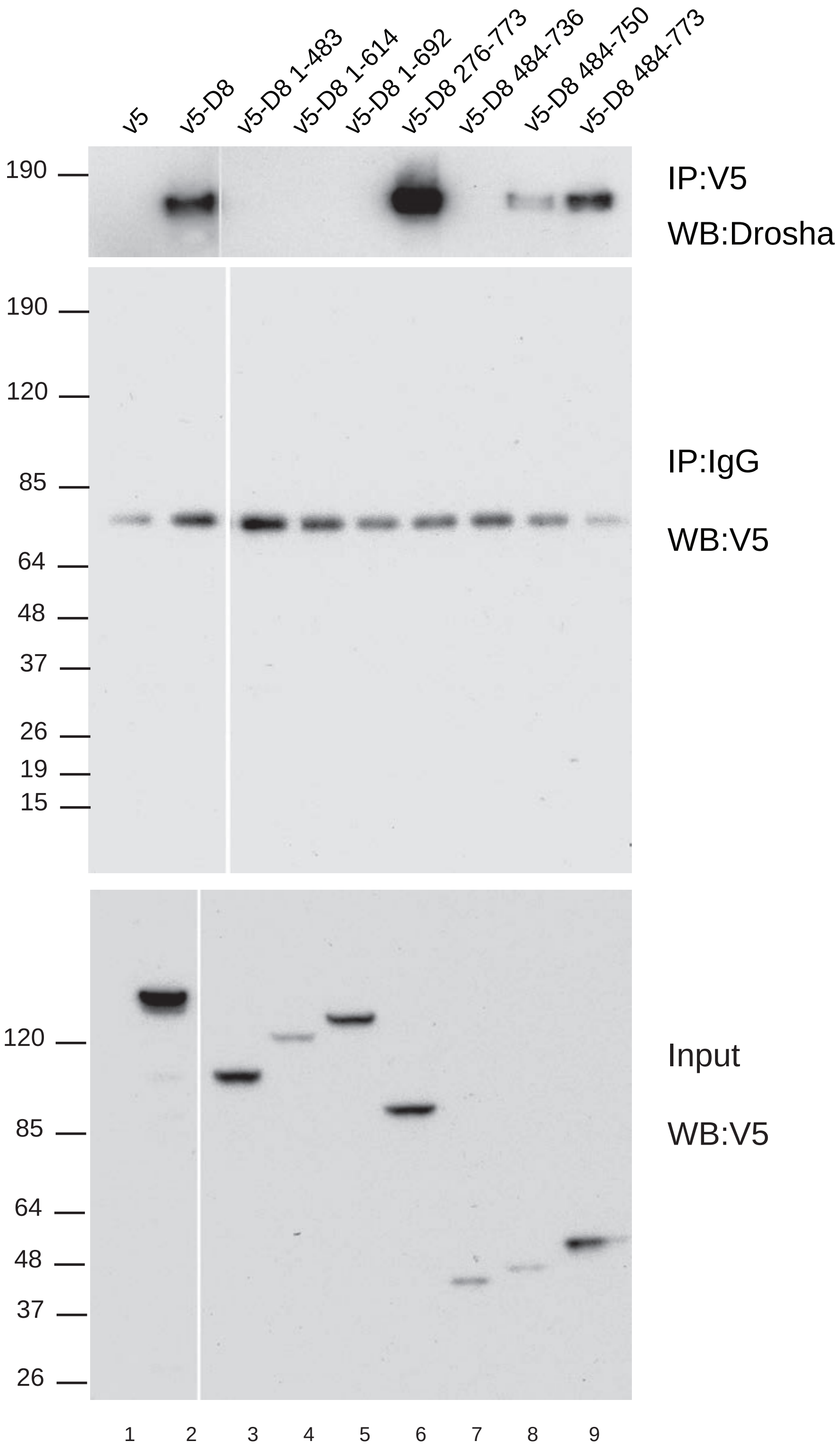

Figure S3, related to Figure 3. Drosha interacts with the C-terminal region of DGCR8. V5-tagged wild-type DGCR8 (V5-D8, lane 2), an empty plasmid control (V5, lane 1), and the indicated DGCR8 truncations (numbers represent amino acid positions, lanes 3-9) were transfected in HEK293Ts and subjected to V5 affinity purification, followed by Western blot with anti-Drosha antibody (top panel). Inputs are shown in the bottom panel. The middle panel is an immunoprecipitation control for Figure 3B. Cells transfected with the same plasmids as in S2A were subjected to IgG immunoprecipitation to control for V5-overexpressed proteins unspecific binding to antibodies and beads.

Figure S4

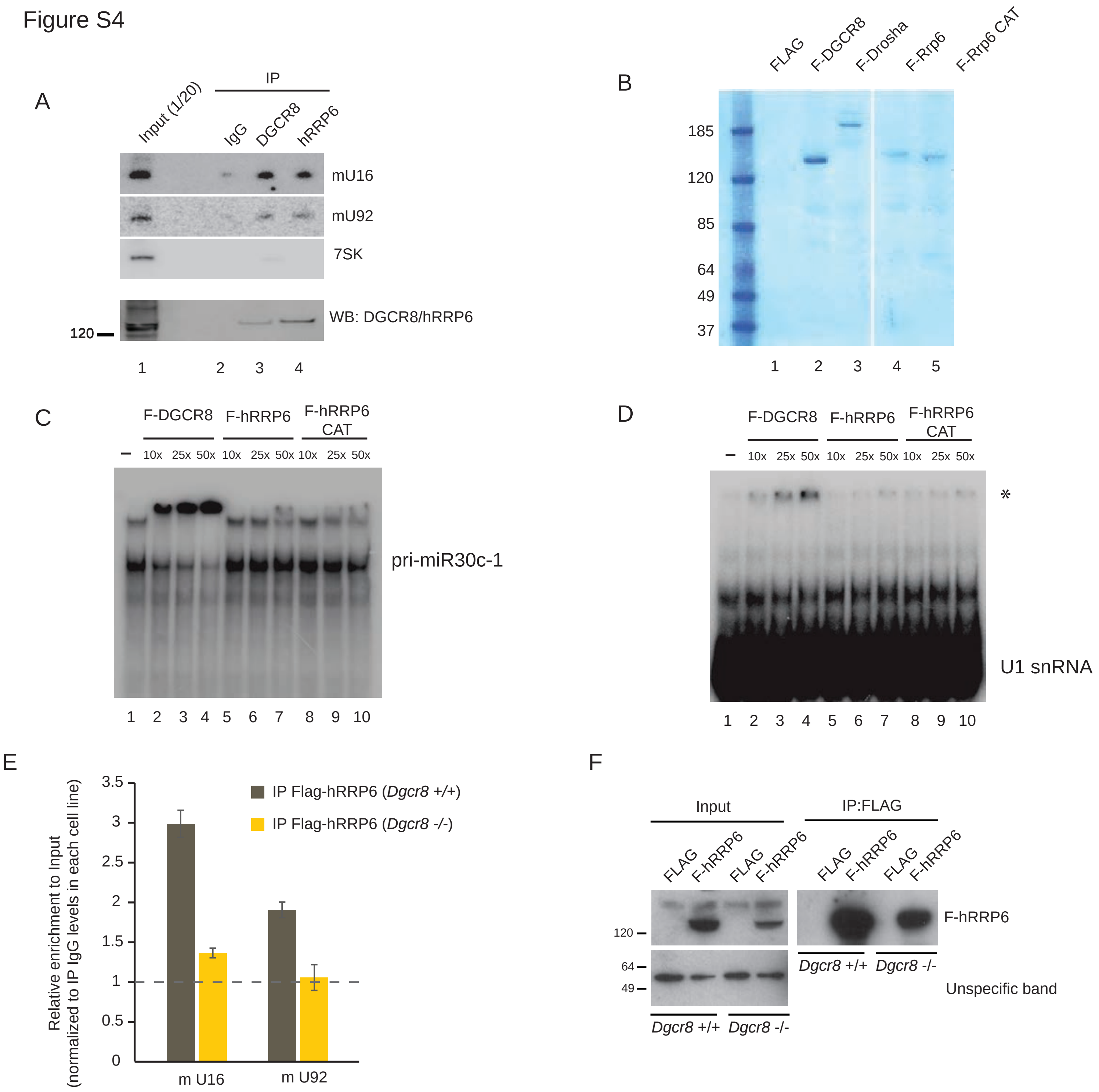

Figure S4, related to Figure 4. DGCR8 promotes hRRP6 binding to mature snoRNAs. (A) Northern blot analyses of the associated mature snoRNAs (mU16 and mU92) to endogenous DGCR8 (lane 3) and hRRP6 (lane 4). IgG immunoprecipitation (lane 2) and 7SK hybridization served as negative controls. Bottom panel, Western blot analyses of immunoprecipitated DGCR8 and hRRP6. (B) Commassie blue staining of FLAG-tagged proteins purified under high stringency conditions. These and similar purifications were used on Figure 4C, S4C and S4D. (C) (D) EMSA analysis of pri-miR-30c-1 (C) and U1 snRNA, as a negative control (D), in the presence of increasing amounts of purified FLAG-DGCR8, FLAG-hRRP6 and FLAG-hRRP6 CAT (D313N, catalytically dead mutant). The molar excess of protein versus radiolabelled RNA is indicated at the top of the panel (1x corresponds to 2.5 nM of protein and 2.5 nM of radiolabelled RNA), asterisk (*) denotes the well. (E) Analysis of human overexpressed FLAG-hRRP6 association to snoRNAs in mouse embryonic stem cells in the presence (Dgcr8 +/+) or absence (Dgcr8 -/-) of DGCR8 by qRT-PCR. (F) Western blot analyses of immunoprecipitated FLAG-hRRP6 that was used in the experiment shown in S4E.

Figure S5

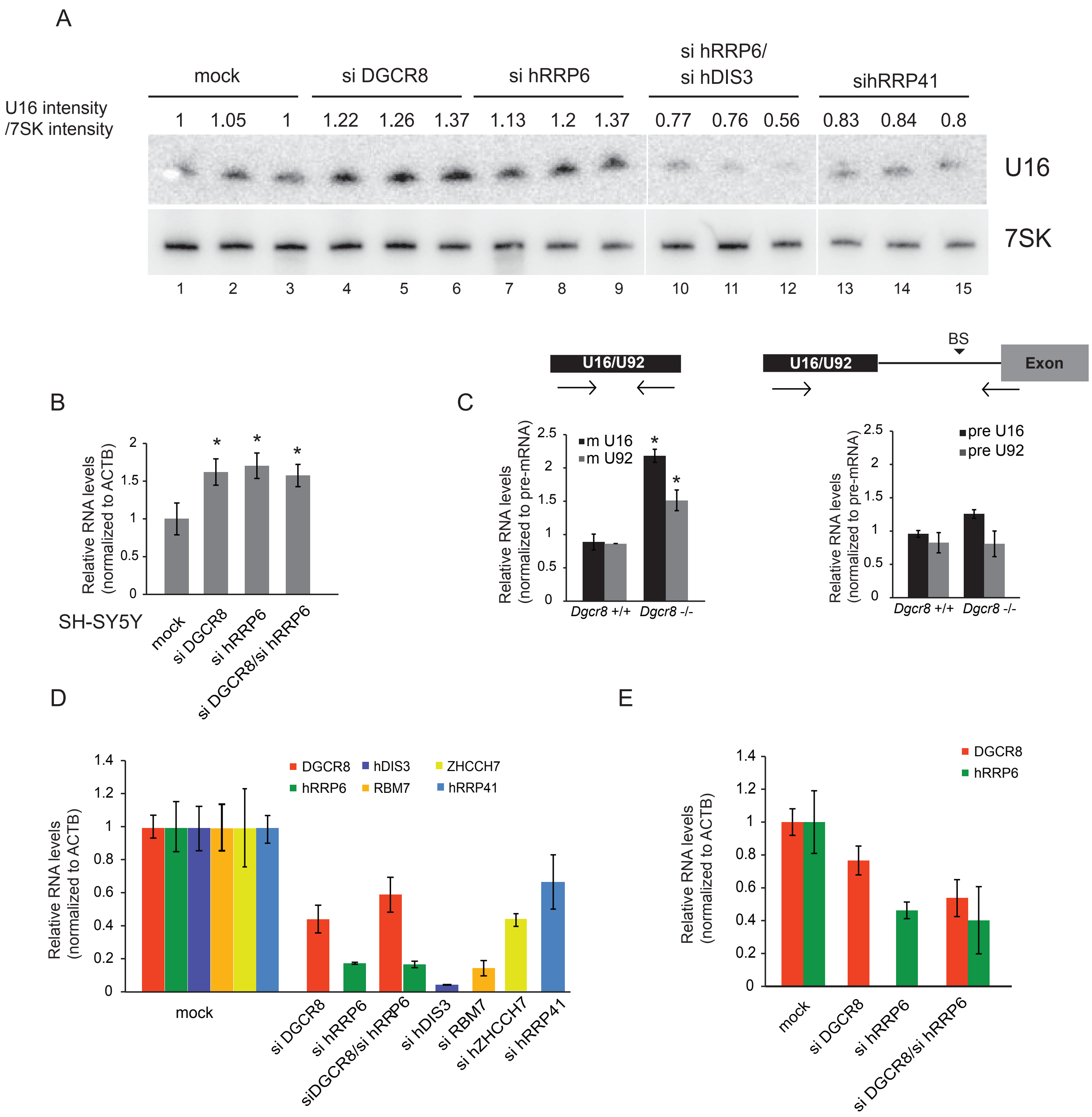

Figure S5, related to Figure 5. SiRNA mediated depletion of DGCR8 and the exosome. (A)Northern blot analyses of U16 snoRNA levels upon depletion of DGCR8, hRRP6, hRRP6/hDIS3 and hRRP41in triplicates in HeLa cells. Quantification values are shown at the top and expressed as the relative intensity normalized to 7SK signal, as a loading control (ImageQuant TL). (B) Quantification of mature U16 levels in SH-SY5Y cells upon transient depletion of DGCR8, hRRP6 and a combination of both. (C) Mature U16 and U92 (left panel) and host pre-mRNA (right panel) levels were quantified in mouse embryonic stem (ES) cells in the presence (Dgcr8 +/+) or absence (Dgcr8 -/-) of DGCR8. (D) RNA levels of DGCR8, hRRP6, hRRP41, hDIS3, ZHCCH7 and RBM7 following siRNA mediated depletion in HeLa cells were analyzed by qRT-PCR. These samples were used on Figures 5B, 5C, S5A and 7D. (E) RNA levels of DGCR8 and hRRP6 followed siRNA-mediated depletion were measured in SH-SY5Y cells by qRT-PCR, these samples were used on Figure S5B and S7A. Values represented in (B) (C) (D) and (E) are the average of at least three biological replicates +/- s.e.m. Asterisks denote significant p-value (≤ 0.05) by Student's t test.

Figure S6

A

snoRNA	Average counts			Fold change (vs. siControl)	
	siControl	sihRRP6	siDGCR8	sihRRP6	siDGCR8
SNORD116-19	84.67	202.67	179.67	2.39	2.12
SNORD116-14	102.33	159.67	171.33	1.56	1.67
SNORD111	20.67	32.00	30.67	1.55	1.48
SCARNA4	48.67	63.67	62.00	1.31	1.27
SNORA19	33.33	52.00	42.33	1.56	1.27
SNORA11	56.67	71.33	71.67	1.26	1.26
snoU13	23.00	27.67	28.67	1.20	1.25
SNORA53	472.00	588.33	567.33	1.25	1.20
SNORA42	41.33	66.00	49.67	1.60	1.20
SNORD116-13	210.00	230.00	252.00	1.10	1.20
SCARNA7	3480.33	3980.33	4128.67	1.14	1.19
SNORD111B	37.33	44.33	43.33	1.19	1.16
SNORA46	58.67	71.33	67.67	1.22	1.15
SNORA80	42.00	52.67	48.33	1.25	1.15
snoU13	23.00	28.33	26.33	1.23	1.14
SNORD6	68.33	98.67	78.00	1.44	1.14
SCARNA5	1412.33	1744.33	1606.33	1.24	1.14
SNORD53	42.67	65.67	47.33	1.54	1.11
SNORD116-7	63.00	79.67	69.33	1.26	1.10

B

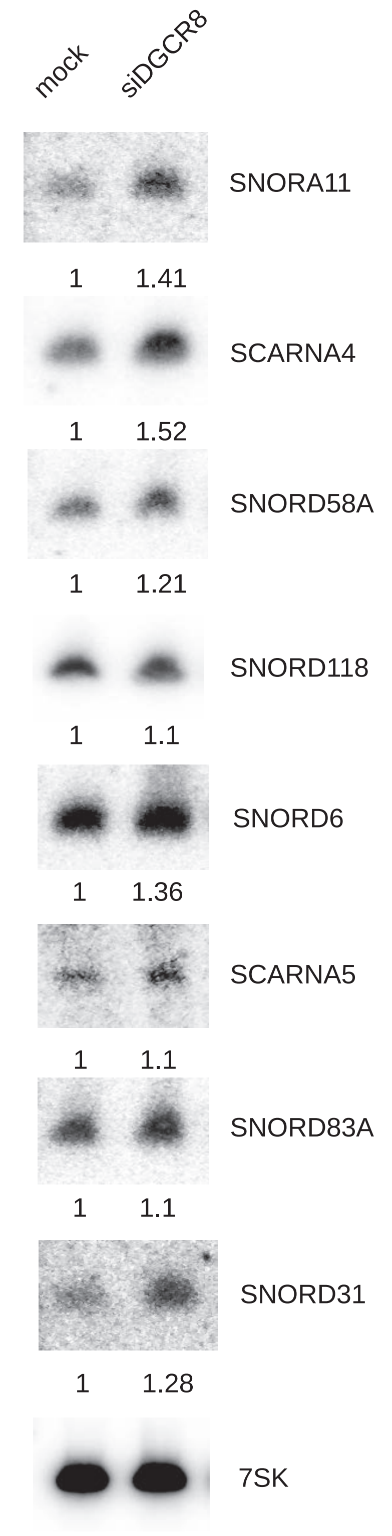

C

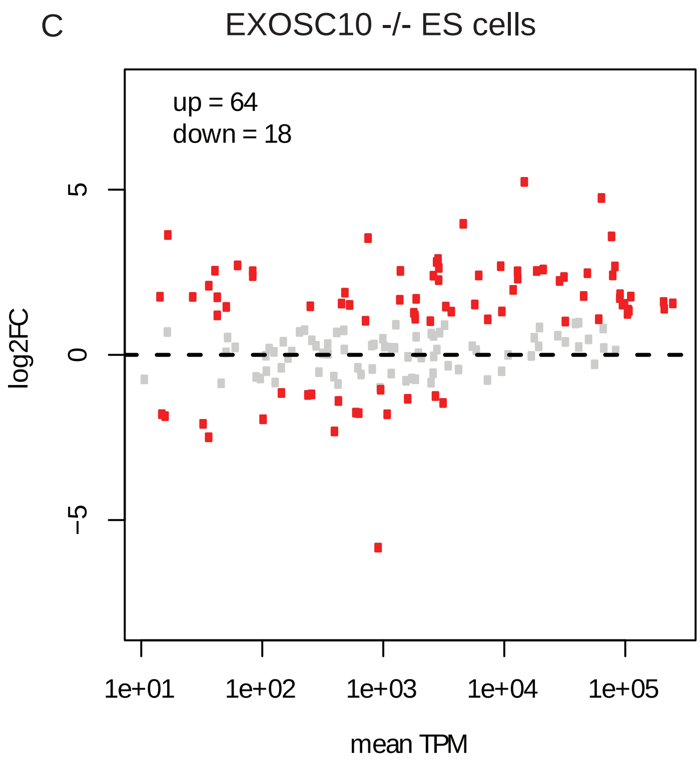

Figure S6, related to Figure 6. DGCR8 and RRP6 control mature snoRNA levels.
(A) Commonly upregulated snoRNAs in RNA-seq data from HeLa cells depleted of DGCR8 (siDGCR8) and hRRP6 (sihRRP6). Average counts are calculated from three biological replicates and fold change is calculated from each condition vs mock depleted cells (siControl). (B) Northern blot analyses of eight different human snoRNAs upon transient depletion of DGCR8 in HeLa cells. Quantification values are shown at the bottom of each gel (ImageQuant TL). 7SK hybridization serves as a loading control. (C) Analysis of snoRNA levels in EXOSC10 KO mouse ES cells (Pefanis et al., 2015). Y-axis shows the log2FC (Fold Change) of snoRNA levels in KO vs. WT cells, and the x-axis is the mean TPM (Tags per million). In red are marked snoRNAs with a log2FC ≥ 1 or ≤ -1. The numbers in the legend represent the number of upregulated (up=64, log2FC ≥ 1) or downregulated (down=18, log2FC ≤ -1) snoRNAs.

Figure S7

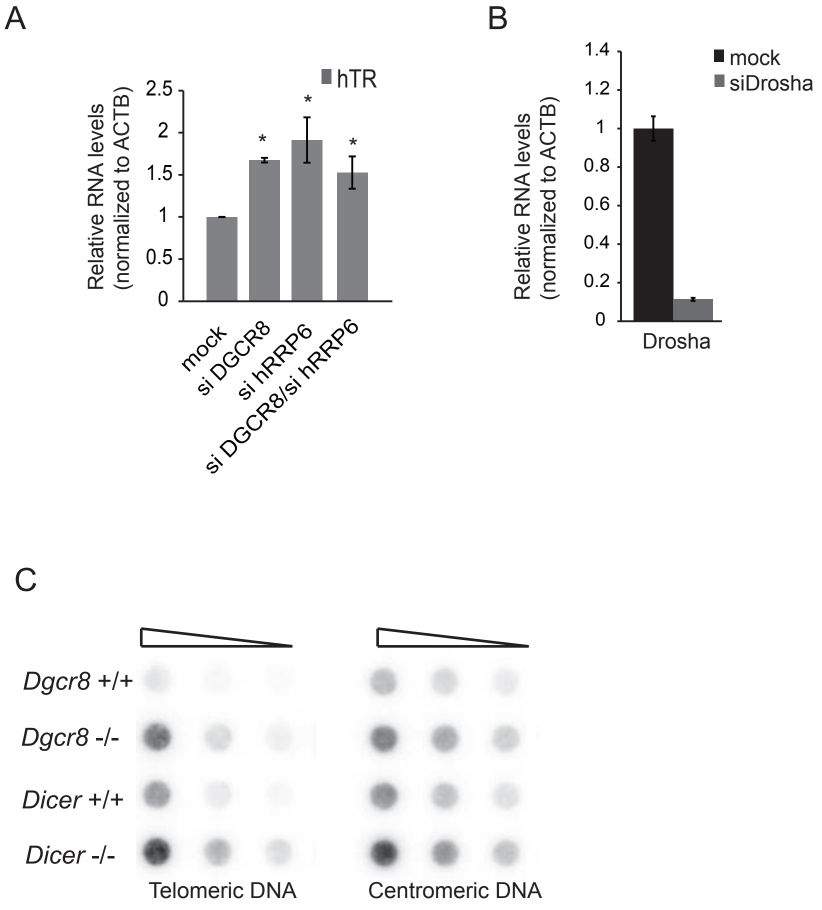

Figure S7, related to Figure 7. DGCR8/exosome complex, but not Drosha, control hTR levels. (A) SH-SY5Y cells were depleted of DGCR8 and hRRP6 and hTR levels were quantified by qRT-PCR (for depletion levels see Figure S5E). (B) The RNA levels of Drosha were quantified by qRT-PCR following siRNA depletion of Drosha in HeLa cells. These samples were used in Figure 7D. Values represented in (B) and (C) are the average of at least three biological replicates +/- s.e.m. Asterisks denote significant p-value (≤ 0.05) by Student's t test. (C) Dot blot analyses of genomic DNA from Dgcr8 -/- and Dicer -/- mouse ES cells and parental cell lines (Dgcr8 +/+ and Dicer +/+) using a telomere repeat probe (TTAGGGx3) and a minor satellite probe as a loading control.

SUPPLEMENTAL TABLES

Table S1. Identification of Factors Interacting with DGCR8 and Drosha by Mass Spectrometry Analyses, Related to Figure 1 (Excel file).

Final list of all proteins identified as interactors of DGCR8 and Drosha.

Table S2. Oligonucleotides Used in this Study, Related to Experimental Procedures.

5'-3' Sequence	Description
GAATTGTCAGGAATTTGCAGTTAACTTGGAGCACCAC	hRRP6 mutagenesis residue D313N
GTGGTGCTCCAAGTTAACTGCAAATTCCTGACAATTC	
TGCCTGCTGTCAGTAAGCTG	human mature U16 qRT-PCR
TGCTCAGTAAGAATTTTCGTCAA	
TGCCTGCTGTCAGTAAGCTG	human precursor (3'end side) U16 qRT-PCR
GGCCTCCACGACACATCTAT	
GTCACCATGCCTCCCTAGAA	human mature U92 qRT-PCR
ATCTGTCTGCCCCGTATCTG	
CGGGACGAATTGAGTGAAAT	human precursor U92 qRT-PCR
AAAGAGGCAGGGCTAAAAGG	
AGCTGAGGCGCTGCTTCT	human pri-miR-24 qRT-PCR
CCTCGGGCACTTACAGACA	
CATCCCCGATAGAGGAGGAC	human 7SK qRT-PCR
GCGCAGCTACTCGTATACCC	
TGGCTCGAATTCCAAGAGTT	mouse host pre-mRNA U16 qRT-PCR
CAGTTGGTCAGTTGCCAAGA	
CTCTGTTCACAGCGACAGTTG	mouse mature U16 qRT-PCR
TTCGTCAACCTTCTGAACCA	
TGGTTCAGAAGGTTGACGAA	mouse precursor U16 (3'end side) qRT-PCR
CCCACGACACATCTGTTTTC	
CCAAGTGCTGGGATTAAAGG	mouse host pre-mRNA U92 qRT-PCR
TGTCCTCAGCACCCTAACAA	
CACTGGACCTCCCCAGAGTA	mouse mature U92 qRT-PCR
AATTGTCTGCCCCGTATCTG	
CAGGGCGAATTGAGTGAAAT	mouse precursor U92 (3'end side) qRT-PCR
GCACAGGGCTGAAAGAAAAA	
GACATCTGTCACCCCATTGA	mouse 7SK qRT-PCR
GCCTCATTTGGATGTGTCTG	
ACAAGGAATTCGCGCCACCCAGTACCCGGGAGCC	hRRP6 cDNA amplification for cloning, containing restriction sites
ACAGTGGCGGCCGCTCCTATCTCTGTGGCCAGTTGTACCTG	
GGCGGTCGGTCGGTGAGGCTTTC	human DGCR8 mRNA qRT-PCR
GGGGCTCTCATCTGTCTCCAT	
ACCCAAGGACCACAGAACAG	human RRP6 mRNA qRT-PCR
TCCAGCAAAAGCCTTGAAGT	

GCTCTGCTTCGAAAACATCC	human DIS3 mRNA qRT-PCR
GCCTGATCCAAAGACTCAGC	
CCCTAACTGAGAAGGGCGTA	hTR RNA qRT-PCR
TGCTCTAGAATGAACGGTGGA	
TCTTAGGACTCCGCTGCC	mouse TERC (mTR) qRT-PCR
CCCACAGCTCAGGTAAGACA	
CGGTTTGTTTGGGTTTGGGTTTGGGTTTGGGTTTGGGTT	mouse telomeric repeat quantification (Callicott 2006)
GGCTTGCCTTACCCTTACCCTTACCCTTACCCTTACCCT	
GAGGGCCAAGTTGGACAGTG	mouse genomic c-myc for normalization (Callicott 2006)
TTGCGGTTGTTGCTGATCTG	
CATGCCCGAACCTACACTG	human Drosha mRNA qRT-PCR
GGTCCTTTCCCACAGCCTAT	
CAACGGATTTGGTCGTATTG	human GAPDH mRNA primers
GGAAGATGGTGATGGGATTT	
TAGTTCAGCGACCTTCAGCA	human hRRP41 mRNA qRT-PCR
GAGGATGGCTGCTTCGAAAG	
TTTCGAGCTTTTCCACCAGG	human RBM7 mRNA qRT-PCR
GTGGGGCATGACTACTTCCT	
GTCATCAGGGAGGAAGAGCA	human ZHCCH7 mRNA qRT-PCR
TCTGACCCATCTGACAGCTG	
TGCTCAGTAAGAATTTTCGTCAA	anti-U16 snoRNA oligo for Northern detection
ATCTGTCTGCCCCGTATCTG	anti-U92 snoRNA oligo for Northern detection
AGGCCATTTTCATTCAGCCC	anti-SNORD6 snoRNA oligo for Northern detection
CAGGCTCACAGCTCAGAAAA	anti-SNORD31 snoRNA oligo for Northern detection
TTCACGGTAAATCCAAAGGTG	anti-SNORD58A snoRNA oligo for Northern detection
CTGTTCTCAGAAGGAAGGCA	anti- SNORD83A snoRNA oligo for Northern detection
AATCAGACAGGAGCAATCAGGGTGTTGCAA	anti-SNORD118 snoRNA oligo for Northern detection
GGGGACGTTTGTTCATAGGG	anti-SNORA11 snoRNA oligo for Northern detection
GTGAAGGCTGCTCTCTCCAA	anti-SCARNA4 snoRNA oligo for Northern detection
GATCATGCACCATCACACCC	anti-SCARNA5 snoRNA oligo for Northern detection
TGCTCTAGAATGAACGGTGGA	anti-hTR oligo for Northern detection
GCGCAGCTACTCGTATACCC	anti-7SK oligo for Northern detection
TTAGGGTTAGGGTTAGGG	anti-Telomere repeat oligo (Prakas Hande et al, Human Mol Genet 2001)
GACTGAAAAACACATTCGTTGGAAACGGGATTTGTAGAAC AGTGTATATCAATGAGTTACAATGAG	anti-Minor satellite mouse oligo (Denis et al, Genes Dev 2001)
CTTGCGAAGAATAAAAAGAAACGAGCTACACTGG	site directed mutagenesis dRBD1 DGCR8
CCAGTGTAGCTCGTTTCTTTTTATTCTTCGCAAG	
GTTGGAAAGCAGTTAAAGAAACAGAAGATCCTTC	site directed mutagenesis dRBD2 DGCR8
GAAGGATCTTCTGTTTCTTTAACTGCTTTCCAAC	
ACAAGGAATTCATGGACTACAAAGACCATGACGG	cloning of FLAG-hRRP6 in pEF1alfa-IRES-RED (containing restriction sites)
TTGTGTCGACCTATCTCTGTGGCCAGTTGTACCTG	

SUPPLEMENTAL EXPERIMENTAL PROCEDURES

Immunoprecipitation and Mass Spectrometry analysis

HEK293T cells were transfected with T7-tagged DGCR8 and pCG backbone as a negative control (for full description see (Macias et al., 2012)). An N-terminus 1x FLAG-DGCR8 (a gift from Sonia Guil), a C-terminus FLAG-Drosha (a gift from Narry Kim) and an empty FLAG control vector were also used. Cells overexpressing DGCR8 or Drosha proteins were collected 48 h after transfection, resuspended in buffer D (20mM Hepes-KOH pH 7.9, 100mM KCl, 0.2mM EDTA, 0.5mM DTT, 0.2mM PMSF, 5% glycerol) and sonicated with Bioruptor for 5 cycles (30 sec on 30 sec off) containing RNAse A or RNAse inhibitors, centrifuged for 10 min at maximum speed, and the supernatant was added to beads overnight at 4°C. For purification of T7-DGCR8, T7 tag antibody agarose from Novagen was used (69026), for FLAG-Drosha and FLAG-DGCR8, anti-FLAG M2 affinity from Sigma (A220) was used. After immunoprecipitation, beads were washed for 5 times with buffer D (150 mM KCl). For mass spectrometry purposes, beads were washed 5 times with buffer D (200 mM KCl). Immunoprecipitated material were loaded in Tris-Glycine gels (Invitrogen) and run for 1-2 cm, so each individual sample was sent as a unique gel slice. The mixture of proteins was analyzed by nLC-MS-MS at the 'FingerPrints' Proteomics Facility (University of Dundee). Proteins that co-purified with FLAG or pCG immunoprecipitations were used as a background for DGCR8 and Drosha purifications. True interactors were defined as those proteins with a mascot score ratios >1 LOG2 (IP mascot score/Control mascot score), and small and large subunit ribosomal proteins were removed from the final list of interactors (see Table S1). For Gene Ontology analysis of the DGCR8-interacting proteins the DAVID Bioinformatic Resources 6.7 was used (Huang et al., 2009). For the purification of endogenous DGCR8 and hRRP6, 1 μg of antibody was coupled to Protein G Dynabeads (10001D, Invitrogen), in the presence of total cell extracts prepared in IP buffer (50mM Tris pH 7.5, 150 mM NaCl, 1mM EDTA and 1% Triton X100).

After overnight binding, beads were washed 5 times, 10 minutes each at room temperature, with IP buffer. For RNA co-immunoprecipitations, RNA was extracted from the beads, as well as from inputs, using Trizol LS (Invitrogen) and following manufacturer's instructions. RNA samples were treated with RQ1 DNAse (Promega, M610A) for 1 h at 37°C, phenol/chloroform extracted and ethanol precipitated. For qRT-PCR experiments, input and immunoprecipitated RNA were quantified using SuperScript III Platinum SYBR Green One-Step qRT-PCR Kit (Invitrogen, 11736-051). The immunoprecipitated RNA was normalized to the input fraction and was expressed relative to the negative control (IgG) (set arbitrarily to 1). Primers used for these analyses are listed in Table S2. For the analysis of native DGCR8 and Drosha complexes, immunoprecipitates from FLAG-DGCR8 and FLAG-Drosha were washed at low salt conditions (150mM KCl-buffer D) and eluted by using 1x and 3x FLAG peptide (F3290 and F4799, respectively) during 6 h at 4°C. Beads were then centrifuged at 8000 g for 1 min, and the supernatant was used to load gradients. For EMSA analysis, FLAG immunoprecipitates were washed at high salt conditions (1M KCl-buffer D) to eliminate associated factors, and eluted using FLAG peptide as described above and quantified using Bradford protein assay. V5-tagged proteins were immunoprecipitated with V5-antibody coated beads (Sigma-Aldrich, A7345), following the same protocol as for endogenous protein immunoprecipitations (IP buffer).

Plasmid construction

FLAG-hRRP6 was amplified from a cDNA clone (Origene) using the primers listed in Table S2 and cloned in a pcDNA3-3xFLAG vector. The FLAG-hRRP6 catalytically inactive mutant (D313N) was generated by Quickchange site-directed mutagenesis kit from Stratagene by mutating the wild-type sequence (FLAG-hRRP6 plasmid) and was based on the mutation described previously (Januszyk et al., 2011). The plasmid overexpressing FLAG-hRRP6 in

mouse ES cells was generated by PCR amplification of the FLAG-hRRP6 ORF and cloning into pEF1alfa-IRES-RED. The mutant DGCR8 expression vector (T7-DGCR8 dRBD1&2 mut) was generated by Quickchange site-directed mutagenesis kit from Stratagene based on (Yeom et al., 2006). For all the primers used for cloning see Table S2.

RNA extraction and quantitative RT-PCR

Total RNA was isolated from cells using Trizol (Invitrogen) and treated with DNAse (RQ1 DNAse, Promega, M601A) and checked for DNA contamination by PCR. For one step qRT-PCR, 500 ng of total RNA was used with SuperScript III Platinum SYBR Green One-Step qRT-PCR Kit (Invitrogen, 11736-051) on CFX96 real time system. Data was analysed with Bio-Rad CFX Manager software. All experiments, unless stated, show the average and standard error of the mean of at least three independent biological replicates. Primers for qRT-PCR analysis are listed in Table S2.

Northern Blot Analysis and probes

Total RNA or RNA extracted after immunoprecipitation of the protein of interest was loaded in a 6% TBE-UREA gel. After running, gel was transferred to Nylon+ membrane (GE Healthcare, RPN119B), and pre-hybridized at 40°C for four hours in Church buffer (1% BSA, 1mM EDTA, 0.5M phosphate buffer, 7% SDS). Radioactive 5'end-labeled oligonucleotides against the RNA of interest were added to the pre-hybridization buffer and incubated O/N at 40°C. First wash was performed with wash buffer 1 (2xSSC, 0.1%SDS) for 1 hour at 40°C, and a second wash with washing buffer 2 (1xSSC, 0.1%SDS) for an additional hour at 40°C. Oligonucleotides used for hybridization purposes are listed in Table S2.

Radioactive RNA labeling and *in vitro* processing reactions

Templates for RNA synthesis and radiolabeling were obtained by PCR containing the T7 promoter as described in (Macias et al 2012). Transcription reactions were performed with T7 polymerase (Ambion, AM2082) in the presence of 40 µmols of ^{32}P-αUTP. Probes were gel-purified, phenol-extracted and ethanol precipitated following standard procedures. Approximately 50,000 cpms of each probe were incubated with 15 ul of immunoprecipitated T7-DGCR8, T7-DGCR8 dRBD1&2 mut, FLAG-Drosha, FLAG-hRRP6, FLAG-hRRP6 mutant and control immunoprecipitate, which were washed with low stringency conditions (150 mM KCl), in the presence of buffer A (0.5mM ATP, 20mM creatine phosphate and 3.2 mM $MgCl_2$). Reactions were incubated for 30 min at 37°C, followed by standard phenol/chloroform extraction and ethanol precipitation. RNAs were resolved in an 8-10% 1xTBE poly-acrylamide urea gel. Gels were analyzed using Phosphorimager (FLA-5100 Phosphorimager Fuji).

EMSA analysis

Reactions contained 40mM Tris-HCl pH 7.9, 6mM MgCl2, 10mM DTT, 10mM NaCl, 2mM spermidine, 0.05% Tween, 20 ug of yeast tRNA, RNase inhibitor, 0.05 pmols of alfa-UTP internally labelled RNA and the same molar quantity (1x) or increasing amounts of the purified protein (FLAG-tagged DGCR8 and Drosha proteins generated by immunoprecipitation in high-washing conditions, 1M KCl, and FLAG-hRRP6 and hRRP6-CAT in 2.5M KCl conditions) and incubated for 20 min at room temperature. Samples were separated in a 6% polyacrylamide (19:1) gels at 200V and exposed in autoradiography films.

Immunofluorescence

Cells were grown on coverslips, fixed with 4% paraformaldehyde, washed with PBS and permeabilized with 0.2% Triton X-100. Coverslips were then incubated for 1 hour with blocking buffer (1% BSA, 0.01% Triton X-100 in PBS). Primary antibodies used to detect nucleolin (MA1-20800, Thermo Scientific) and T7 overexpressed proteins (Merck-Millipore, 65922); and secondary antibodies used were anti-rabbit Alexa Flour® 488 (A11070, Molecular Probes) and anti-mouse Alexa Flour® 594 (A21203). All antibodies were diluted to working concentrations in diluted blocking buffer (1:1000) and incubated with coverslips in humidified chamber overnight at 4°C. Coverslips were washed 3 times with wash buffer (0.01% Triton X-100 in PBS). Secondary antibodies were incubated with coverslips in a dark, humidified chamber for 2 hours at room temperature. Coverslips were then washed with washing buffer and mounted with DAPI containing mountant. Images taken on Ziess Axioplan2 with an objective lens mounted to a PIFCO collar. Images were deconvolved and processed using Volocity.

Nucleolar purification

Ten 150mm plates of HeLa cells were collected by trypsinization when ~90% confluent. Nucleolar purification was essentially carried out as previously described (http://www.lamondlab.com/f7nucleolarprotocol.htm) with one modification; S3 buffer was supplemented with 0.1mM $MgCl_2$. Antibodies employed as nucleoplasmic markers were anti-Lamin B (ab16048, Abcam) and anti-eIF4A3 (ab115022, Abcam); and as a nucleolar marker anti-fibrillarin (ab4566-250, Abcam).

Dot blots for telomere repeat quantification

Different amount of genomic DNA (1ug, 500 ng and 250 ng) was diluted in a solution containing 0.4M NaOH and 10mM EDTA and denature at 100°C for 10 min. After cooling on ice, samples were applied to a Hybond N+ membrane using a Manifold vacuum system. Membrane was previously rinsed in water. After DNA was applied to the membrane, a wash step with 0.4M NaOH was performed. The membrane was finally washed twice with 2xSSC and air-dried. For detection of telomere repeats, membrane was pre-hybridized overnight at 40°C in Church buffer (1% BSA, 1mM EDTA, 0.5M phosphate buffer and 7% w/v SDS). The oligonucleotide used for detection of telomere repeats was 5'end labelled with T4 PNK (M0201, NEB) following the manufacturer's instructions. The radiolabelled oligonucleotide was then added to the pre-hybridization solution and incubated overnight at 40°C. Membrane was after washed at 40°C for 2 times during 30 min with wash buffer 1 (2XSSC, 0.1% SDS) and once for 30 min with wash buffer 2 (1xSSC, 0.1%SDS). Dot blots were analyzed using Phosphorimager (FLA-5100 Phosphorimager Fuji).

Telomere length analysis

Average telomere length was measured by real time quantitative PCR from genomic DNA extracted from *Dgcr8* +/+, *Dgcr8* -/-, *Dicer* +/+ and *Dicer* -/- cell lines following a previously described protocol (Callicott and Womack, 2006). Primers used for this experiment are listed in Table S2.

Analysis of DGCR8 HITS-CLIP and hRRP6-iCLIP libraries

Reads were pre-processed using custom python scripts. First, reads were demultiplexed according to their fixed barcode allowing up to 1 mismatch. Next, reads were trimmed to remove low quality scores and 3' adapter sequences. Finally, duplicated reads containing

identical random barcodes were removed and the 5' random barcodes were trimmed. After these steps, all reads longer than 19 nucleotides were further analysed. Reads were mapped to the human genome (hg19) using bwa-pssm (Kerpedjiev et al., 2014). All reads that were not confidently mapped (posterior probability PP <= 0.99) were then mapped to an exome index containing all collapsed exons from human ensembl70 transcripts (Flicek et al., 2014). Finally, all reads unmapped to the genome were mapped to an exon-junction index containing all annotated unique exon-junctions from human ensembl70 transcripts. Only reads mapped at any of the steps with a PP > 0.99 were considered for further analysis.

Reads from the 2 CLIP biological replicates were pooled and clustered together according to their genomic positions. Significant clusters were calculated using Pyicos (Althammer et al., 2011) and only significant clusters with a false discovery rate (FDR) < 0.01 were considered for further analysis. DGCR8 significant clusters were obtained from (Macias et al., 2012) (http://regulatorygenomics.upf.edu/Data/DGCR8/). We only downloaded the data of the second replicate both for the endogenous and the overexpressed experiments. For each of the datasets, the coordinates of the significant clusters were mapped from hg18 to hg19 using liftOver tool (Kuhn et al., 2013). To define a set of reproducible DGCR8 CLIP clusters, T7-DGCR8 and endogenous DGCR8 significant clusters were overlapped according to their genomic coordinates using fjoin (Richardson, 2006). DGCR8 reproducible clusters and hRRP6 significant clusters were overlapped based on their genomic coordinates using fjoin. These common clusters were then overlapped with the ensembl70 annotation using fjoin to identify a set of target genes bound by both DGCR8 and hRRP6.

Standardized snoRNA profiles

To make standardized profiles, annotated SNORD, SCARNA and SNORA were with at least 30% RNA-seq coverage in HEK293 cells were selected from ensembl70 annotation. The RNA-

seq used to calculate the RNA-seq coverage of the snoRNAs was a pool of published RNA-seq datasets from (Baltz et al., 2012; Kishore et al., 2011) (GSM714684; GSM714685; GSM940576), which were mapped using the same pipeline described above.

To make the profiles, each annotated snoRNAs was divided in 20 equally sized bins. We also included 50nt at each flank of the annotated snoRNA, which were divided in 5 equally sized bins. In each bin we calculated the mean enrichment per nucleotide of iCLIP $\bar{e}_b$ for bin b as

$$\bar{e}_b = \frac{1}{g}\sum_{i=1}^{g}\frac{1}{l_i}\sum_{j=1}^{l_i}\frac{e_i}{10^6}$$

where g is the total number of snoRNAs considered, l_i is the length of the bin and e_i is the number of iCLIP reads mapped to the bin.

Analysis of hRRP6 and DGCR8 knockdown RNAseq data

The total RNA from three biological replicates for each knockdown condition was sequenced using Illumina HiSeq2500s. The reads were trimmed using fastx-trimmer (fastx version 0.0.13) with options -f 11 -l 110 -Q 33. Reads were mapped to the human hg19 (GrCh37) genome, using tophat version 2.0.10, with the options --library-type fr-firststrand -r -50 --mate-std-dev 40. Raw reads counts were generated using Ensembl 75 (GrCh37.p13) genes annotation and featureCounts (from the subread package, version 1.4.6) with the options -s 2 –p.

Analysis of EXOSC10 KO mouse embryonic cells data

Processed RNA-seq data from wild type and *Exosc10* KO mouse embryonic cells (mESC) was obtained from Pefanis et al., (2015) as BigBed files containing TPM for each condition (courtesy from the authors). For each condition, TPM values were overlapped with snoRNA

and scaRNA annotation from Ensembl67 (Flicek et al., 2014) using fjoin (Richardson, 2006). For each gene, the average TPM value in each condition was calculated.

SUPPLEMENTAL REFERENCES

Althammer, S., Gonzalez-Vallinas, J., Ballare, C., Beato, M., and Eyras, E. (2011). Pyicos: a versatile toolkit for the analysis of high-throughput sequencing data. Bioinformatics *27*, 3333–3340.

Baltz, A.G., Munschauer, M., Schwanhäusser, B., Vasile, A., Murakawa, Y., Schueler, M., Youngs, N., Penfold-Brown, D., Drew, K., Milek, M., et al. (2012). The mRNA-bound proteome and its global occupancy profile on protein-coding transcripts. Mol. Cell *46*, 674–690.

Callicott, R.J., and Womack, J.E. (2006). Real-time PCR assay for measurement of mouse telomeres. Comp. Med. *56*, 17–22.

Flicek, P., Amode, M.R., Barrell, D., Beal, K., Billis, K., Brent, S., Carvalho-Silva, D., Clapham, P., Coates, G., Fitzgerald, S., et al. (2014). Ensembl 2014. Nucleic Acids Res *42*, D749–D755.

Huang, D.W., Sherman, B.T., and Lempicki, R.A. (2009). Systematic and integrative analysis of large gene lists using DAVID bioinformatics resources. Nat. Protoc. *4*, 44–57.

Januszyk, K., Liu, Q., and Lima, C.D. (2011). Activities of human RRP6 and structure of the human RRP6 catalytic domain. RNA *17*, 1566–1577.

Kerpedjiev, P., Frellsen, J., Lindgreen, S., and Krogh, A. (2014). Adaptable probabilistic mapping of short reads using position specific scoring matrices. BMC Bioinformatics *15*, 100.

Kishore, S., Jaskiewicz, L., Burger, L., Hausser, J., Khorshid, M., and Zavolan, M. (2011). A quantitative analysis of CLIP methods for identifying binding sites of RNA-binding proteins. Nat. Methods *8*, 559–564.

Kuhn, R.M., Haussler, D., and Kent, W.J. (2013). The UCSC genome browser and associated tools. Brief. Bioinform. *14*, 144–161.

Macias, S., Plass, M., Stajuda, A., Michlewski, G., Eyras, E., and Cáceres, J.F. (2012). DGCR8 HITS-CLIP reveals novel functions for the Microprocessor. Nat. Struct. Mol. Biol. *19*, 760–766.

Pefanis, E., Wang, J., Rothschild, G., Lim, J., Kazadi, D., Sun, J., Federation, A., Chao, J., Elliott, O., Liu, Z.-P., et al. (2015). RNA Exosome-Regulated Long Non-Coding RNA Transcription Controls Super-Enhancer Activity. Cell *161*, 774–789.

Richardson, J.E. (2006). fjoin: simple and efficient computation of feature overlaps. J. Comput. Biol. *13*, 1457–1464.

Yeom, K.-H., Lee, Y., Han, J., Suh, M.R., and Kim, V.N. (2006). Characterization of DGCR8/Pasha, the essential cofactor for Drosha in primary miRNA processing. Nucleic Acids Res. *34*, 4622–4629.
